# The use of the extended reality technologies in simulation-based health professions education: a bibliometric analysis

**DOI:** 10.3389/fmed.2026.1787542

**Published:** 2026-04-13

**Authors:** Afaf Sulaiman Alblooshi, Falah Mohammed Almarzooqi, Marwa Gaffar Alameen, Taleb Mohamed Almansoori, Latifa Nasser Alderei, Aryam Mohammed Albreiki, Gamila Ahmed, Saif Al-Shamsi, Faten Abdullah AlRadini

**Affiliations:** 1 Department of Medical Education, College of Medicine and Health Sciences, United Arab Emirates (UAE) University, Al Ain, Abu Dhabi, United Arab Emirates; 2 Department of Family Medicine, College of Medicine and Health Sciences, United Arab Emirates (UAE) University, Al Ain, Abu Dhabi, United Arab Emirates; 3 Department of Radiology, College of Medicine and Health Sciences, United Arab Emirates (UAE) University, Al Ain, Abu Dhabi, United Arab Emirates; 4 College of Medicine and Health Sciences, United Arab Emirates (UAE) University, Al Ain, Abu Dhabi, United Arab Emirates; 5 Public Services and Outreach Unit, National Medical Library, College of Medicine and Health Sciences, United Arab Emirates (UAE) University, Al Ain, Abu Dhabi, United Arab Emirates; 6 Department of Internal Medicine, College of Medicine and Health Sciences, United Arab Emirates (UAE) University, Al Ain, Abu Dhabi, United Arab Emirates; 7 Department of Family and Community Medicine, College of Medicine, Princess Nourah bint Abdulrahman University, Riyadh, Saudi Arabia

**Keywords:** augmented reality, clinical simulation, immersive learning, mixed reality, simulation in medical education, simulation-based education, virtual reality

## Abstract

**Introduction:**

Extended Reality (XR), encompassing Virtual Reality (VR), Augmented Reality (AR), and Mixed Reality (MR), represent an increasing advancement in simulation-based Health Professions Education (HPE) by enabling immersive, learner-centered training that improves motivation, knowledge retention, and skill acquisition and development. In recent years, XR has gained significant attention as an innovative tool in HPE, offering interactive and experiential learning opportunities. Despite growing interest, a comprehensive understanding of research trends, influential contributions, and thematic developments remains limited.

**Methods:**

Publications on Extended Reality Technologies in simulation-based HPE from 2010 to July 29, 2025, were retrieved from Web of Science (WoS) Core Collection, Scopus, and PubMed. Bibliometric analyses and network visualizations were conducted using Biblioshiny in R and Microsoft Excel to describe publication growth, collaboration patterns, influential sources and authors, citation impact and keyword trends.

**Results:**

A total of 2,789 publications from 1019 journals, authored by 12,886 researchers affiliated with 4,276 institutions from 88 countries, were included. Annual publication output increased markedly after 2018. Research productivity was concentrated in high-income countries, led by the United States and China, while contributions from Low- and Middle-Income Countries (LMIC) remained limited. The University of Toronto was the most productive institution. Author productivity was concentrated among a small group of leading contributors, including Konge L, Ahmed K, and Dasgupta P. *Clinical Simulation in Nursing* and the *Journal of Surgical Education* were the most frequent outlets. Keyword analysis revealed dominant themes including virtual reality, simulation training, surgical education, and clinical competence, with increasing representation across nursing, residency, and interdisciplinary education contexts.

**Conclusion:**

XR simulation-based HPE research is rapidly expanding and increasingly collaborative field, driven by technological innovation and evolving competency-based educational models, but scholarly output remains geographically concentrated. Future research should prioritize theory-driven implementation, equitable and context-sensitive adoption, and longitudinal evaluation of educational outcomes across diverse health professions and settings.

## Introduction

Technological innovations are rapidly transforming all fields, with education experiencing particularly significant change (1, 2). The shift from traditional tools such as pens, pencils, and textbooks to interactive digital platforms has fundamentally altered how knowledge is delivered and acquired (3). In medicine, technology-enhanced learning is increasingly used to simplify complex scientific and clinical information, enabling students, practitioners, and researchers to visualize and interact with concepts in more meaningful ways (4–6). These digital tools, and virtual platforms replicate classroom settings, provide safe, controlled spaces to conduct simulations and experiments that may carry risks in real life (7). Their adoption across Health Professions Education (HPE) reflects a broader effort to promote learning outcomes through engagement, accessibility, and interactivity (8).

Extended Reality (XR), an innovative simulation-based learning modality, is a term that encompasses Virtual Reality (VR), Augmented Reality (AR), Mixed Reality (MR), and other computer-generated environments utilizing head-mounted displays (HMDs) (9, 10). XR offers cost-effective, immersive, and highly adaptable simulation-based learning experiences (10, 11). VR has been integrated into HPEand practice for nearly three decades, especially for advanced 3-Dimensional (3D) visualization (4, 12, 13). More recently, AR and MR have emerged as valuable adjuncts in healthcare and clinical specialties such as neurosurgery, psychiatry, and neurology, and in medical education (4, 13, 14). While all three modalities fall under the XR umbrella, each provides a distinct user experience:

- VR offers complete immersion in a virtual environment, replacing all real-world stimuli with computer-generated input via HMDs (4, 15).
- AR overlays digital content onto the real-world view, enabling simultaneous interaction with both physical and virtual elements through smartphones or HMDs (4, 16, 17).
- MR blends real and virtual environments along a continuum, allowing users to manipulate and engage with virtual objects as though they exist in the physical world (4, 18).

In HPE, XR technologies are increasingly being applied to support simulated learning by simulating clinical scenarios in safe, controlled learning environments. XR can enhance traditional simulated learning, which typically involves task trainers, standardized patients, and mannequins (19). Traditional simulation remains essential for providing multisensory, hands-on technical performance, and complex patient interaction (20). Accordingly, XR-based simulation is designed to complement rather than replace in-person simulation; by enabling additional, self-paced practice outside formal timetabled teaching sessions, it can extend and reinforce skills developed through traditional SBME. In contrast, XR offers cost effective, immersive, and interactive learning experience that can be accessed more flexibly and frequently than facility-based simulation (21–24). XR-enabled simulations provide low-risk environments where learners can practice critical clinical skills, navigate complex cases, and engage with ethical decision-making (25, 26). These environments also support repeated practice, which can reinforce mastery and confidence before learners engage in high-stakes or resource-intensive fields (27–29).

XR can broaden opportunities for skill reinforcement and active learning by complementing traditional simulation-based education. Through immersive 3D simulations and interactive models, learners can explore real-world scenarios without leaving the classroom, increasing retention and motivation (10, 30). These tools enable educational experiences that are not possible in conventional settings, such as manipulating virtual anatomical structures or interacting with responsive clinical simulations (31). Despite its rapid adoption and growing promise, important research gaps persist. Most existing studies focus on isolated disciplines or short-term educational outcomes, with limited longitudinal evidence on how XR influences long-term clinical competence or patient care (10). Furthermore, comparative research examining the relative effectiveness of VR, AR, and MR remains limited. This gap is exacerbated by the pace of technological advancement, which often exceeds academic investigation (32).

XR has the potential to transform medical education across all levels of training. Its expanding range of applications emphasizes the need for ongoing research and integration into evidence-based educational frameworks. This study presents a comprehensive bibliometric analysis of current literature on the use of XR in simulation-based HPE. Specifically, it seeks to identify publication trends, influential authors and institutions, collaborative networks, and key thematic areas. It also highlights existing research gaps and opportunities for theory-driven innovation and future development.

## Methods

### Study design

This study used a bibliometric analysis approach, to quantitatively examine the Use of Extended Reality Technologies in simulation-based HPE. Bibliometric indicators were applied to evaluate the publication trends, identify leading contributors, and conduct productivity and frequency analyses.

### Data sources and search strategy

**FIGURE 1:**
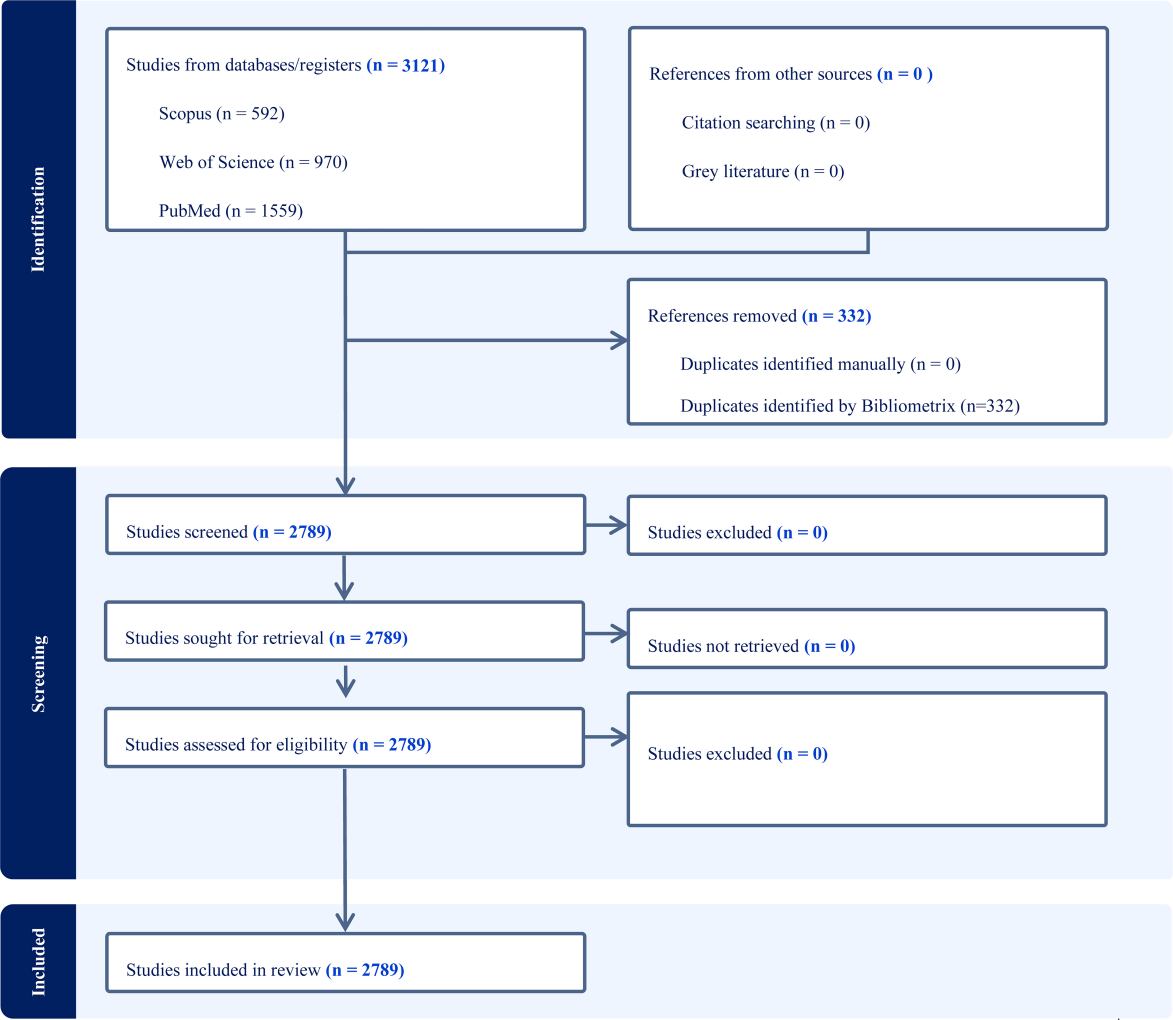
Flow diagram illustrating database identification, record merging, duplicate removal, and construction of the bibliometric dataset.

Data were extracted from three major citation databases with comprehensive coverage: Web of Science (WoS) Core Collection, Scopus, and PubMed on July 29th, 2025, by a medical librarian (GA) (Figure 1). The search strategy was developed using Medical Subject Headings (MeSH) and title/abstract fields to ensure comprehensive literature retrieval. Keywords related to AR, VR, and MR simulation-based medical education included:

- “Simulation-based Medical Education” OR “Medical Simulation” OR “Simulation Training”
- “Immersive technology”
- “Virtual reality” (VR) OR “Augmented reality” (AR) OR “Mixed reality” (MR) OR “Extended reality” (XR)
- “XR in Healthcare”
- “AR OR VR OR MR”
- Head-Mounted Display (HMD)
- Health Professions Education
- Clinical Education
- Medical Training

Search strategies were customized for each database, with an English language filter applied. A detailed description of the search terms and strategy is provided in Supplementary Material 1. Additionally, reference lists of included studies were manually screened for further eligible articles; however, no additional records were included through this step. Gray literature was excluded to maintain the quality and rigor of the analysis. The search was restricted to articles published between 2010 and 2025, ensuring coverage of recent advancements in XR in simulation-basedHPE. Only peer-reviewed journal articles and conference proceedings were included, while book chapters, editorials, and non-peer-reviewed sources were excluded.

### Eligibility criteria

Inclusion criteria:

- Studies explicitly focus on XR in simulation-based medical education in undergraduate, graduate, or continuing medical education.
- Research addressing trends, applications, or innovations in simulation-based training.
- Publications which are indexed in Web of Science, Scopus, and PubMed with full citation records available.

Exclusion criteria:

- Non-English publications.
- Studies not related to XR in simulation-based learning in medical education.
- Editorials, commentaries, and non-peer-reviewed papers.
- Studies on non-medical fields or unrelated simulation training.

To validate dataset relevance and minimize inclusion of non-XR simulation literature, a dataset specificity audit was conducted on the Scopus-exported records (*n* = 592). XR-related terms (VR/AR/MR/XR) were present in the title, abstract, or author keywords in 589 records (99.49%). A random manual review of 50 records confirmed that 49/50 (98%) were explicitly focused on XR applications in simulation-based HPE.

### Data synthesis and analysis

Quantitative bibliometric analyses were performed using the Biblioshiny (the web interface of the Bibliometrix package, version 5.1.0) in R (version 4.4.1; R Foundation for Statistical Computing, Vienna, Austria) alongside Microsoft Excel version 365 for data management and descriptive summaries. Using Biblioshiny the following variables were extracted and analyzed: publication year (to describe trends over time), citation count (to assess scholarly impact), authors and affiliations (to identify leading contributors and institutions), source journals (to determine high-impact research publication outlet), and author keywords and thematic trends (to characterize the evolving research focus over time).

All bibliographic records from WoS, Scopus, and PubMed were imported into Biblioshiny and merged using the built-in “Merge Datasets” function with default settings. During this process, duplicates were automatically identified (primarily using DOI and title/year matching) and collapsed into a single entry. When the same document was indexed in more than one database, Biblioshiny retained a single record according to its default database priority (WoS, followed by Scopus, then PubMed). Citation analyses were then performed on this merged dataset using the total citations field provided by the retained source record. All bibliometric indicators reported in this study were therefore calculated from the fully deduplicated merged dataset.

To address potential author and institutional name variants across databases, manual data cleaning was performed on the merged dataset prior to final analysis. Author names with minor variations in spelling were harmonized into a single standardized form after verification against publication titles and affiliations. Institutional names were similarly standardized where clear spelling or abbreviation differences existed, while distinct entities were preserved. To illustrate key research trends and identify potential knowledge gaps, the following analyses were conducted: Publication trend (Growth of research output over time), co-authorship analysis (mapping collaboration networks across authors, institutions, and countries), influence and productivity (top journals and authors), keyword co-occurrence analysis (identifying emerging themes, topic evolution, and research gaps), and citation analysis (highlighting the most influential publications and authors in simulation-based learning research), and Country/institution map (Global distribution of research contributions).

In addition, a thematic evolution analysis was conducted using the Thematic Evolution module in Biblioshiny to examine conceptual changes over time. Two time periods (2010–2021 and 2022–2025) were defined to allow comparison of thematic changes.

For network analyses, visualizations were generated in Biblioshiny using the automatic layout option. Networks were limited to the top 50 nodes to enhance readability. Edge weights were normalized using the association strength method. Community detection was performed using the Louvain clustering algorithm, and a repulsion force of 0.5 was applied to optimize node spacing in the visualizations.

Keyword based thematic analyses were conducted using author-supplied keywords extracted from the bibliographic records in Web of Science, Scopus, and PubMed. No selective exclusion of author-provided keywords was applied to preserve the original structure of the data and ensure reproducibility of the bibliometric outputs. The thematic structure of the literature was explored using keyword co-occurrence analysis visualized through a word cloud generated using the Biblioshiny package. Highly frequent terms reflect common author keyword and indexing practices and were therefore interpreted in context with emphasis placed on conceptually meaningful educational themes relevant to XR Simulation-based HPE research.

## Results

### Analysis of bibliometric data overview

A total of 2,789 documents were retrieved from 1,019 sources between 2010 and 2025, demonstrating an annual growth rate of 12.48% and a strong trend toward multi-author and internationally collaborative research (Table 1).

**TABLE 1:** Main information about data.

Timespan	2010:2025
Sources (journals, books, etc.)	1,019
Documents	2,789
Annual growth rate %	12.48
Document average age	6
Average citations per doc	10.71
Cited references	0
Document contents	
Keywords plus (ID)	5,781
Author’s keywords (DE)	4,969
Authors	
Authors	12,167
Authors of single-authored docs	58
Authors’ collaboration	
Single-authored docs	61
Co-authors per doc	5.76
International co-authorships %	16.24

### Analysis of publication trend

**FIGURE 2:**
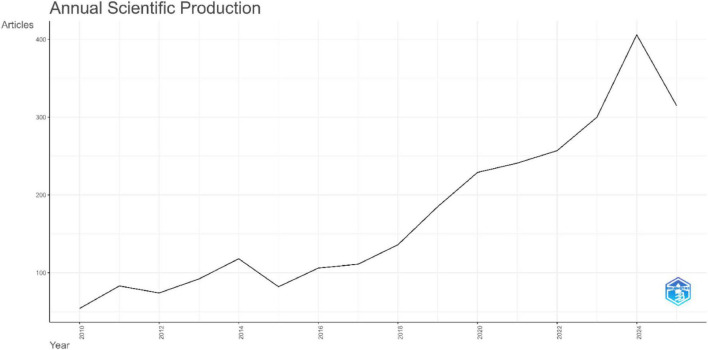
Analysis of publication trend: annual scientific production.

**FIGURE 3:**
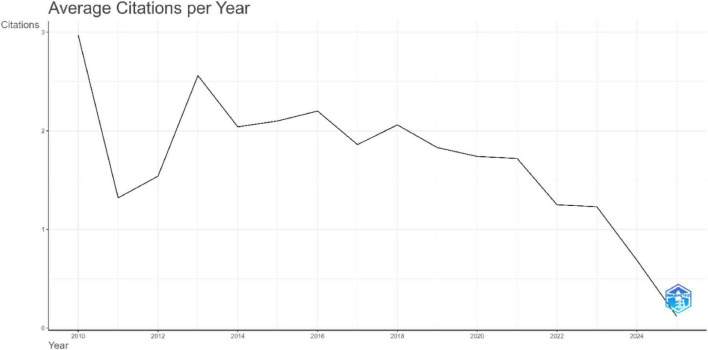
Analysis of publication trend: average citation per year.

The annual number of publications outlined in Figure 2 shows a consistent steady upward growth over the past 15 years, beginning with 54 articles in 2010 and rising steadily, with more pronounced growth from 2018 onward. Output increased from 136 publications in 2018 to a peak of 406 in 2024. In 2025, 315 publications were recorded; however, this lower value reflects data collection only up to July and may not represent the full annual total. This growth underscores the increasing academic engagement and continuing ongoing expansion of research activity on XR applications in HPE. The number of citations per year reveals the highest mean citations per article for the publications between 2010 and 2014, with 2010 articles averaging 47.56 citations (Figure 3). Citation averages declined over time, reflecting both the increasing number of publications and the shorter period available for recent works to accrue citations. More recent articles published from 2023 onward show lower averages, largely due to limited citable years and the shorter time available for citations to accumulate.

### Analysis of countries and institutions

**FIGURE 4:**
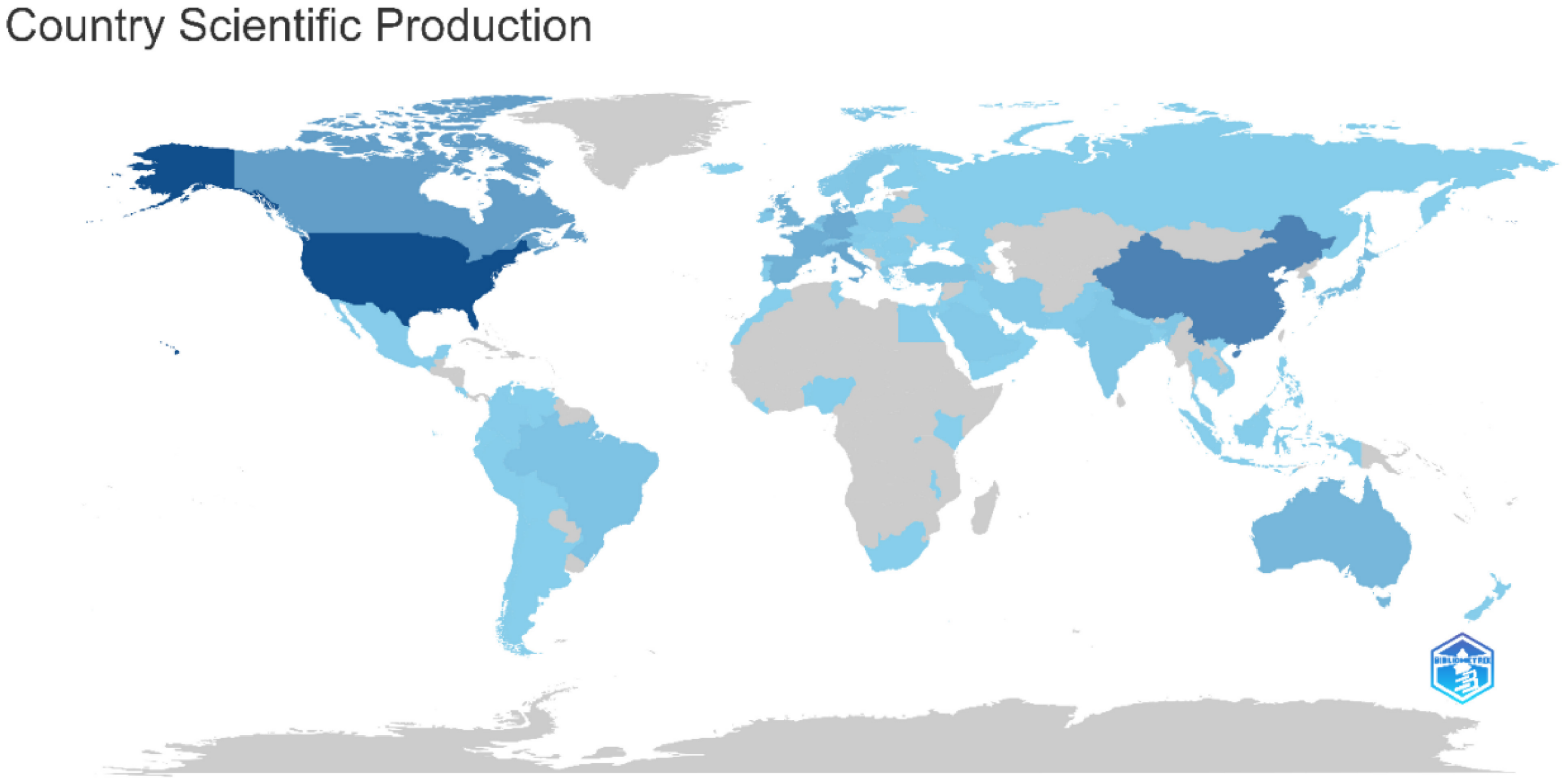
Country scientific production.

**FIGURE 5:**
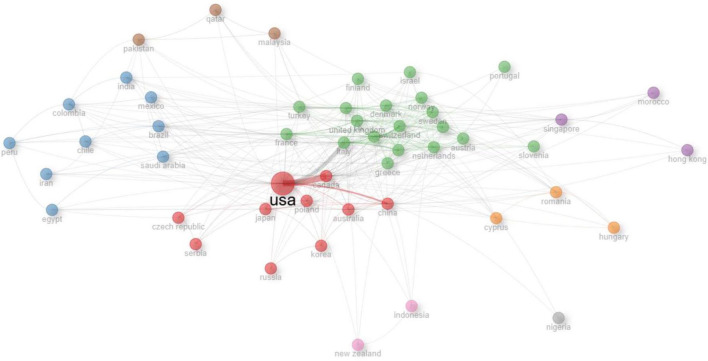
Countries’ collaboration network.

A total of 88 countries were found to contribute to the scientific production of articles related to the Use of Extended Reality in simulation-based HPE (Supplementary Material 8). Country level production was dominated by the United States, China, and Canada, followed by several European countries, reflecting concentrated global research activity in this area (Figure 4 and Supplementary Material 8). The global map of scientific country production further illustrates this pattern, with darker shading observed in high-output countries such as the United States, China, and Canada, and lighter shading in regions with lower levels of publication activity. A focus analysis of Gulf Cooperation Council (GCC) country scientific production shows that Saudi Arabia leads the region’s research output (83 articles), followed by Qatar (21 articles). Oman and the United Arab Emirates contribute more modestly (7 and 6 articles, respectively), while Kuwait has the lowest output (2 articles) among the GCC countries. Overall, the data highlight that scientific production in the region is concentrated primarily in Saudi Arabia, with varying levels of contribution from other members (Supplementary Material 9). Figure 5 illustrates global research collaborations based on co-authorship. Node size reflects publication volume, and edge thickness indicates collaboration strength (normalized by association). Three major clusters are identified: North America, East Asia, and Central Europe (red), Western/Northern Europe including Turkey (green), and Latin America, Middle East, and South Asia (blue). Smaller clusters and peripheral nodes show regional or specialized collaborations. The United States of America (USA) is the central hub with strong bilateral ties to Canada, China, and the United Kingdom. This pattern reflects strong productive global integration, with leading nations forming dense collaborative ties and emerging economies contributing increasingly to the international research landscape.

**FIGURE 6:**
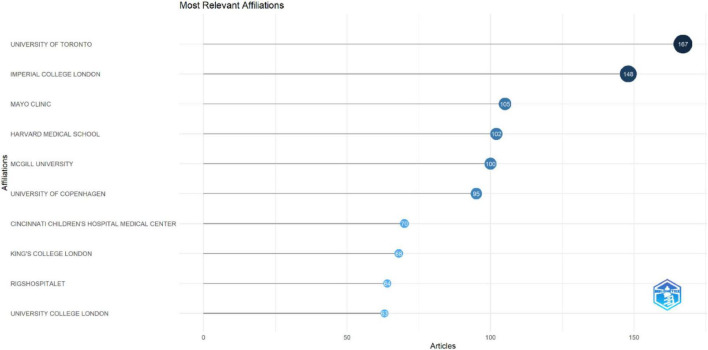
Most relevant affiliation.

The analysis of the leading institutional affiliations showed that most productive institutions were led by the University of Toronto, followed by major contributions from universities in the United Kingdom, the United States, and Canada (Figure 6). The distribution reflected strong institutional engagement from both North America and European regions, highlighting the growing global focus on integrating XR technologies into HPE.

### Analysis of authors and cited authors

**FIGURE 7:**
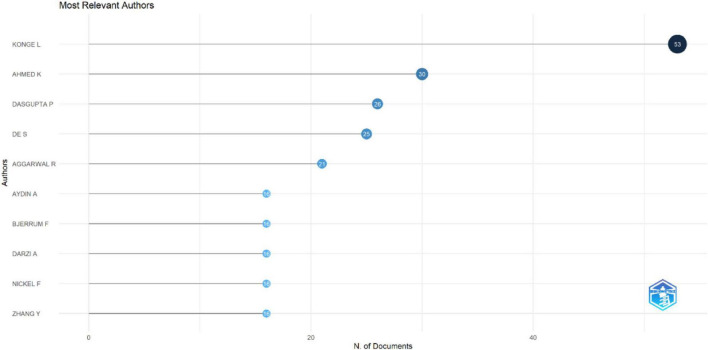
Most relevant authors.

The leading authors, based on the number of publication output in XR technologies within HPE, are summarized in Figure 7. Overall, author productivity was concentrated among a small group of highly active contributors, led by Konge L, reflecting strong leadership and collaborative research networks in advancing research on the use of XR technologies in the field of HPE.

**FIGURE 8:**
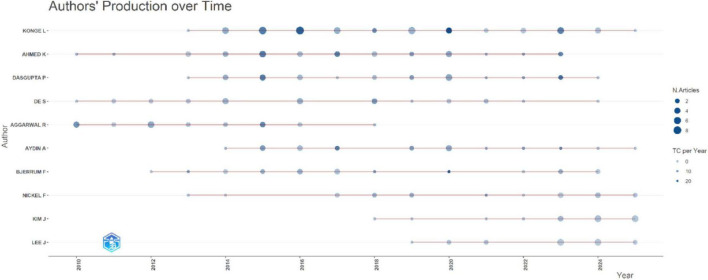
Authors’ production over time.

The analysis of author production over time revealed varying trends in publication activity and citation impact among the most productive contributors in the field Figure 8. Author output increased notably after 2015, with several contributors demonstrating sustained productivity and rising citation influence over time. Konge L maintained the most consistent publication activity across the study period, while Ahmed K and Dasgupta P also demonstrated sustained output with periods of higher citation counts. More recent increase in activity was observed by authors such as De S and Aggarwal R. In Figure 8, bubble size represents the number of articles published per year, while the color intensity reflects total citations per year. Overall, the trend shows a gradual broadening of the authorship base, alongside strengthening collaborative activity and growing citation impact in more recent years of the dataset.

**FIGURE 9:**
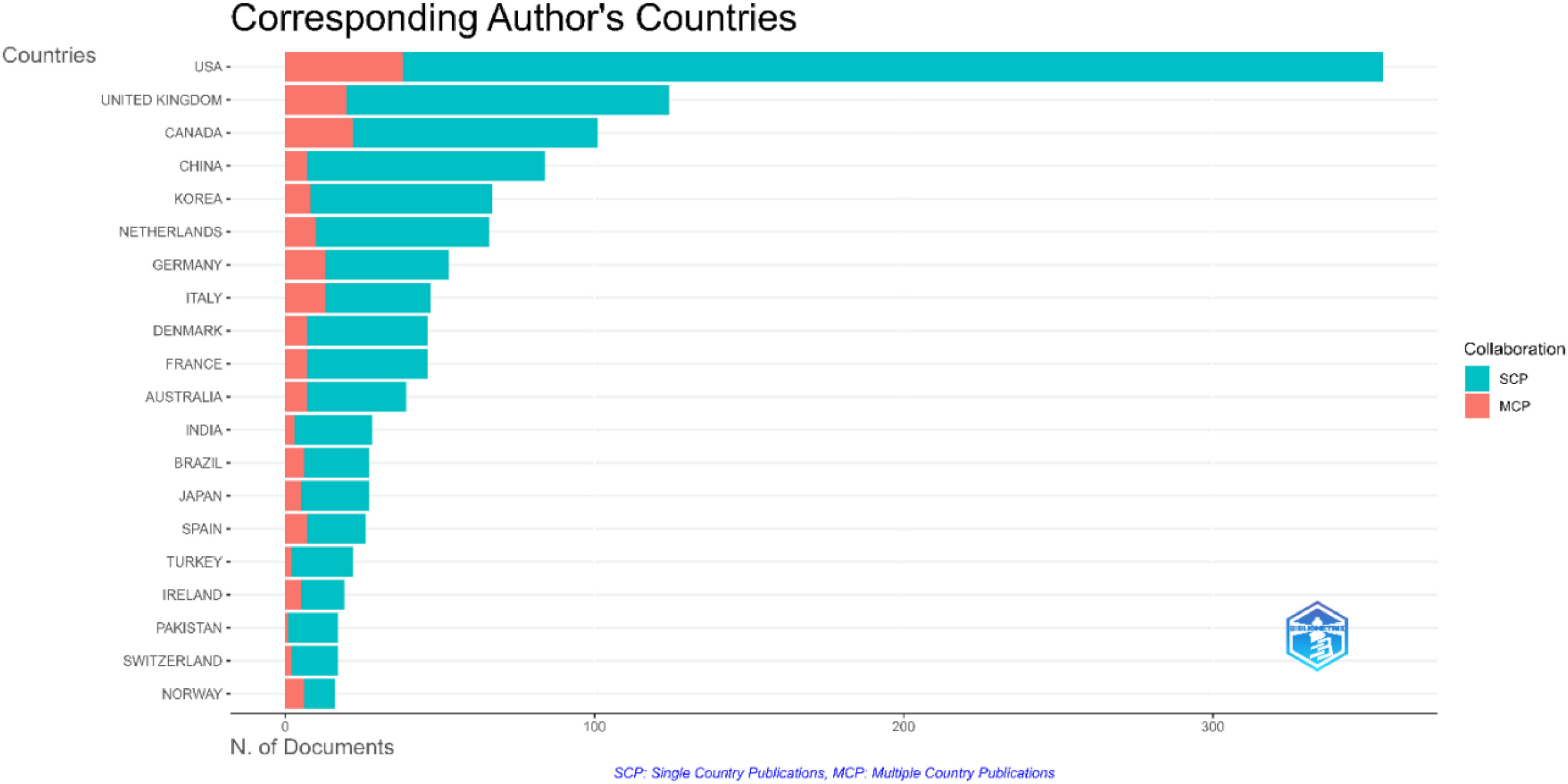
Corresponding authors’ countries.

The analysis of the corresponding authors countries revealed that the United States had the highest publication output, with 355 articles (12.73%). Most of these were single country publications (SCP, *n* = 317), while 38 articles (10.7%) were multi-country publications (MCP) indicating that majority of the United States output was produced without international co-authorship. The United Kingdom (124, 4.45%) and Canada (101, 3.62%) followed, with higher MCP rates (16.13 and 21.78%, respectively), reflecting greater engagement in international collaborative research. Other major contributions included China (84, 3.01%), Korea (67, 2.40%), and the Netherlands (66, 2.37%). Germany, Italy, Spain, and Ireland showed comparatively high MCP percentages (24.53–37.50%), reflecting international collaboration despite lower total publication counts (Figure 9).

### Analysis of documents/sources

The analysis of the collected sources reveals key patterns and trends that provide a basis for understanding the scope and focus of the sources. Table 2 shows the top 10 relevant journals contributing to publication in the field. The analysis of these sources reveals that research on the topic is concentrated in a set of specialized journals, with Clinical Simulation in Nursing the most frequently represented journal in the dataset (*n* = 91), reflecting its strong focus on simulation-based educational methods in healthcare. This is followed closely by the Journal of Surgical Education (*n* = 82) and Surgical Endoscopy and Other Interventional Techniques (*n* = 78), reflecting a significant interest from surgical education and minimally invasive surgery communities. Other notable sources included BMC Medical Education (*n* = 51), Journal of Medical Internet Research (*n* = 49), and Nurse Education Today (*n* = 41). Lower but still relevant representation was observed in Surgical Endoscopy (*n* = 36), World Neurosurgery (*n* = 36), Studies in Health Technology and Informatics (*n* = 30), and BMJ Open (*n* = 28) highlight a diverse publication base across open-access and specialized journals. This distribution shows that research on XR technologies in Simulation-based HPE is most prominently published in journals specializing in simulation, surgical training, and technology-enhanced medical education, reflecting the multidisciplinary nature of the field.

**TABLE 2:** Most relevant sources.

No.	Sources	Articles
1	Clinical Simulation in Nursing	91
2	Journal of Surgical Education	82
3	Surgical Endoscopy and Other Interventional Techniques	78
4	BMC Medical Education	51
5	Journal of Medical Internet Research	49
6	Nurse Education Today	41
7	Surgical Endoscopy	36
8	World Neurosurgery	36
9	Studies in Health Technology and Informatics	30
10	BMJ Open	28

### Analysis of reference

The most globally cited documents represent foundational works that have shaped subsequent research on XR in simulation-based HPE. Table 3 summarizes the five most frequently cited publications in the dataset, including total citations, citations per year, and normalized total citation. Normalized Total Citation was used to reduce citation bias related to publication age by standardizing citation impact based on citations per year, enabling comparison across older and more recent publications. The distribution of highly cited works includes early simulation-focused scholarships such as Lateef F in the Journal of Emergencies, Trauma, and Shock, as well as more recent studies including Li L in the American journal of Translational Research that demonstrate high citation rates relative to publication age. The presence of foundational simulation-based medical education (SBME) papers reflects their frequent citation within XR-related publications included in the dataset, rather than inclusion of non-XR records. As citation metrics capture influential references within the retrieved corpus, highly cited conceptual SBME framework papers may appear due to their central role in shaping XR-related research. Overall, the citation distribution reflects a combination of highly cited foundational publications and more recent studies with strong annual citation impact.

**TABLE 3:** Most global cited documents.

No.	Paper	Title	DOI	Total citations	Total citation per year	Normalized total citation
1	Lateef F, 2010, J EMERG TRAUMA SHOCK	Simulation-based learning: Just like the real thing	10.4103/ 0974-2700.70743	563	35.1875	11.83878505
2	Van H P D, 2010, BRIT J SURG	Objective assessment of technical surgical skills	10.1002/bjs.7115	376	23.5	7.906542056
3	Hamstra S J, 2014, ACAD MED	Reconsidering fidelity in simulation-based training	10.1097/ ACM.0000000000000130	363	30.25	14.83685487
4	Li L, 2017, AM J TRANSL RES	Application of virtual reality technology in clinical medicine	PMC5622235*	320	35.55555556	19.06602254
5	Kotsis S V, 2013, PLAST RECONSTR SURG	Application of the “see one, do one, teach one” concept in surgical training	10.1097/ PRS.0b013e318287a0b3	271	20.84615385	8.137075718

*The article by Li et al. (2017) has no assigned DOI; therefore, the PMC version was cited to ensure a stable reference.

### Analysis of emerging themes and research directions

**FIGURE 10:**
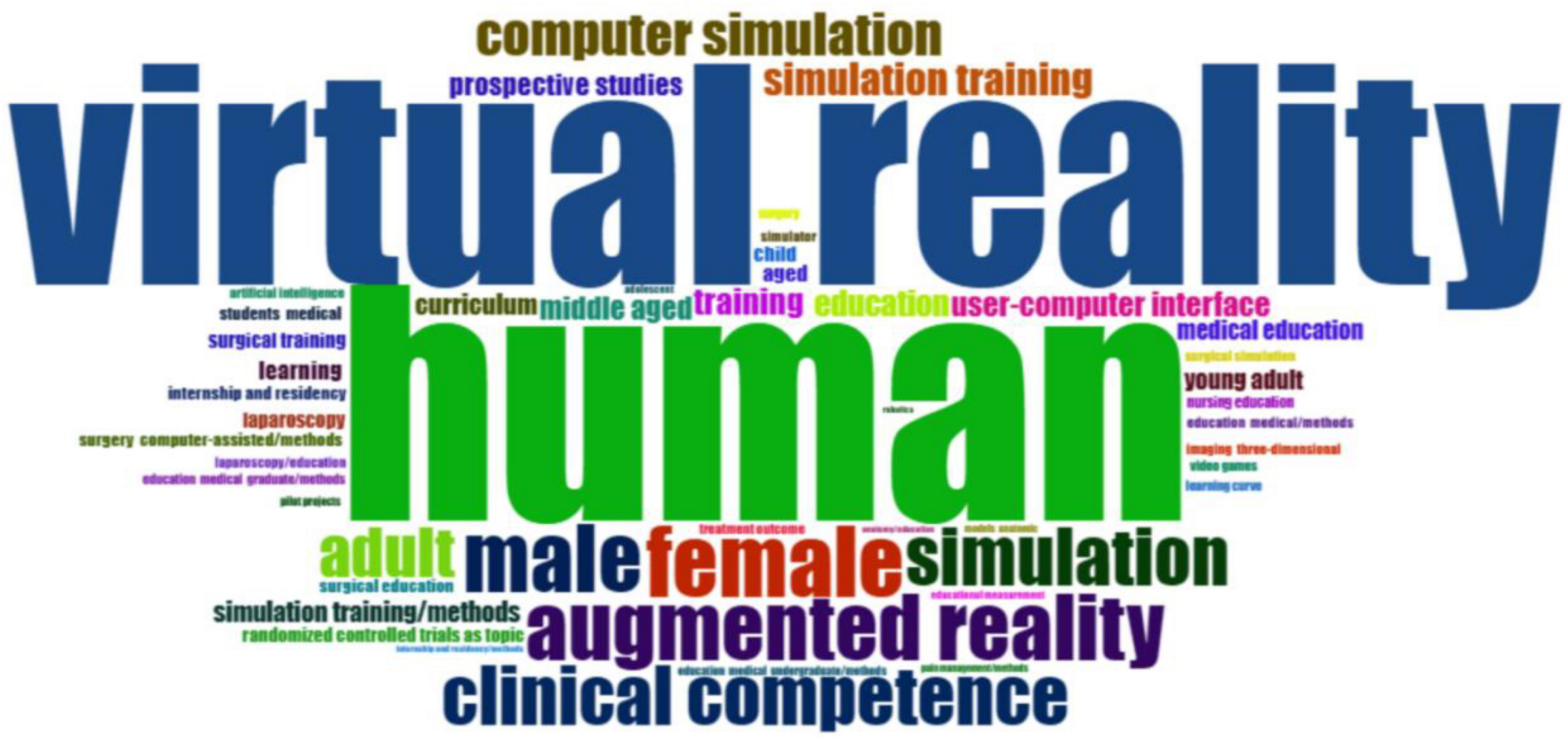
Word cloud.

The word cloud visualization (Figure 10) illustrates the most frequently occurring keywords extracted from the bibliometric dataset. Several high-frequency terms (e.g., “human,” “male,” and “female”) present common and descriptive practices within bibliographic records rather than standalone educational themes and were therefore interpreted cautiously. Beyond these descriptions, the visualization highlights the prominent concentration of conceptually meaningful keywords related to immersive learning and simulation-based training, including *“virtual reality,” “augmented reality,” “simulation,” “simulation training,”* and *“clinical competence.”* Procedural terms (e.g., *“laparoscopy” and “surgical training”*) and emerging technological integration such as “artificial intelligence” were also observed.

**FIGURE 11:**
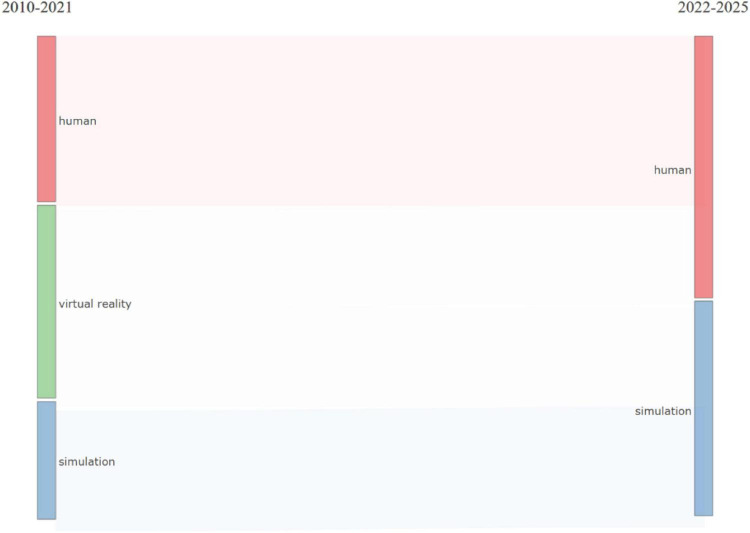
Thematic evolution (time-sliced).

Thematic evolution analysis (Figure 11) demonstrates relative continuity in core concepts across time periods. Between 2010 and 2021, dominant themes centered on “virtual reality,” “simulation,” and human-focused descriptors. In the more recent period (2022–2025), “human” and “simulation” remained prominent within the thematic structure.

**FIGURE 12:**
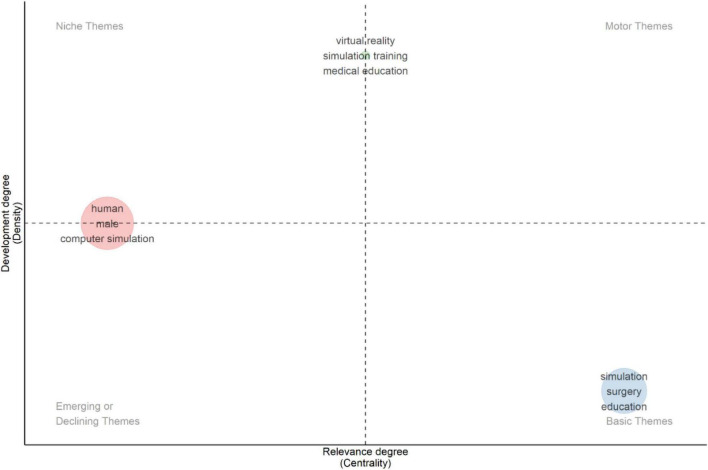
Strategic thematic map: period 2010–2021.

**FIGURE 13:**
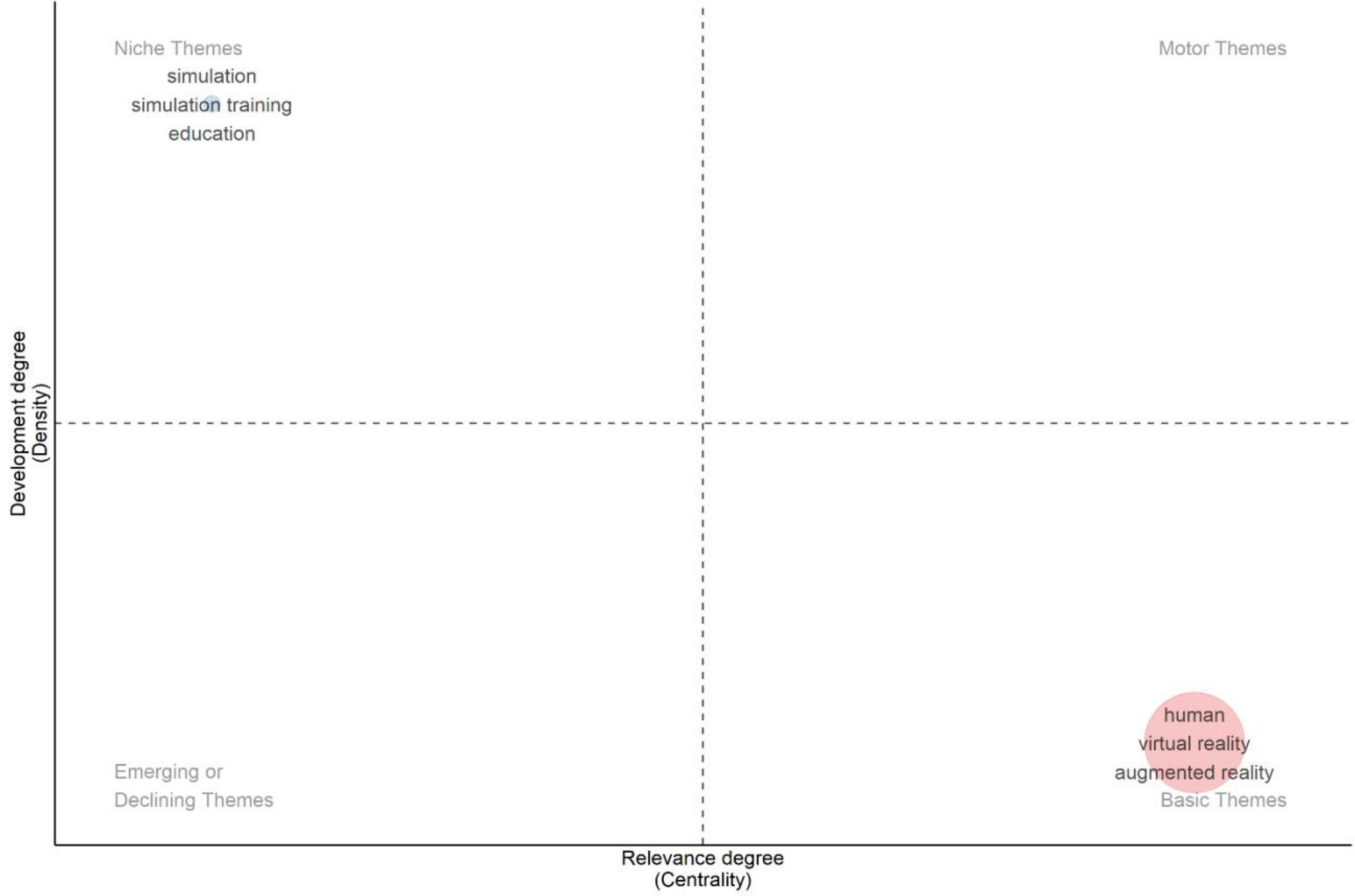
Strategic thematic map: period 2022–2025.

Strategic thematic mapping was performed for two time periods (2010–2021 and 2022–2025) using centrality (relevance) and density (development) to classify themes into four quadrants: niche, motor, emerging/declining, and basic themes (Figures 12, 13). This strategic thematic mapping across the two time periods (2010–2021 and 2022–2025) revealed structural shifts in XR-related HPE scholarship. During 2010–2021, Virtual reality, simulation training, and medical education were positioned as niche themes (high density, low centrality). In contrast basic theme terminology (simulation-surgery-education) shows high centrality within the field but lower internal density. Human, male, and computer simulation were in emerging/declining themes, reflecting limited structural integration despite frequent keyword occurrence. No motor themes were identified.

For the 2022–2025 period, simulation training and education remained a niche theme, demonstrating continued internal development. However, a notable shift occurred in the basic quadrant, where human, virtual reality, and augmented reality formed a highly central cluster, highlighting greater thematic convergence and integration of immersive modalities. No prominent themes were identified within the emerging/declining or motor quadrant. Overall, the results shows sustained emphasis on immersive simulation technologies with increasing thematic consolidation in recent years.

## Discussion

In this research, bibliometric analytical measures were conducted on the relevant literature addressing the Use of Extended Reality technologies in simulation-based HPE. The focal points for this analysis were publication and author scores, as well as a trend analysis. Overall, research has been steadily increasing, with a recent spike in publications, underlining the relevance of research on XR applications in HPE.

### Publication trends and research growth

The global annual publication volume has been increasing over the past 15 years, with recent pronounced growth noted from 2018 onward. The steady increase in annual publications observed in our analysis underscores the growing academic knowledge and engagement with XR technologies use in HPE (33). This trend reflects the broader expansion of immersive and interactive technologies across health professions education, where VR, AR, and MR have gained increasing attention as tools to enhance learning, assessment, training, and simulation. Comparable trajectories have been reported in several studies, all of which report a marked accelerating growth of XR research outputs over the past decade across medical, healthcare, surgical training, and educational contexts to help develop professional skills (34–37). This expansion is likely driven by advances in affordable XR technologies, the growing emphasis on competency-based education, and the surge in digital adoption during the COVID-19 pandemic (10, 38, 39). Together, these trends are positioning XR as an increasingly established area of scholarship. Nonetheless, key gaps persist, as research output is dominated by high-income countries, particularly the United States and China, with limited contributions from low- and middle-income regions (LMIC) (10). This imbalance may reflect structural barriers to XR implementation and evaluation, including high costs, limited access to XR devices and supporting infrastructure, and evolving regulatory and ethical frameworks (33, 40). Additional concerns related to data privacy, governance and professional accountability, especially in Artificial Intelligence (AI) supported applications may further limit institutional uptake and research activity in these settings (33). Moreover, studies often focus on feasibility rather than standardized outcome evaluation, limiting comparability, while rapid technological advances risk fragmenting the evidence base (10, 32). Addressing these gaps will require future development of cost-sensitive, context adaptive XR solutions and blended educational models that integrate XR with established simulation approaches (33). Strengthened regulatory and collaborative research initiatives and rigorous, longitudinal evaluation will also be needed to support broader global participation and equitable integration of XR in HPE (33, 40).

Although the annual productivity was increased but the study revealed that citation averages declined over time, reflecting both the increasing number of publications and the shorter period available for recent works to accrue citations (41). Then in more recent years from 2023 onward, lower averages were largely due to limited citable years and the shorter time available for citations to accumulate (42).

### Global and institutional contributions

The United States and China have the dominance in XR-related publications in HPE, with substantial contributions from several European countries and Canada. These findings are consistent with previous bibliometric studies (34, 36, 43, 44). Across bibliometric analyses, the United States remains the leading contributor, while China demonstrates rapid growth. This expansion may be associated with national strategies promoting VR and immersive technology development (36). European countries and Canada provide complementary outputs, collectively reinforcing the central role of the United States and China in shaping XR scholarship (34, 36, 43, 44). These findings reflect a broader trend toward adopting XR technologies to create effective learning environments for healthcare professionals and highlight global interest in XR for advanced training and skill development (45). While publication volume is concentrated in the United States and China, their comparatively low proportion of international co-authorship (10.7 and 3.01%, respectively) suggests a stronger domestic orientation than countries such as the United Kingdom and Canada (16.13 and 21.78%, respectively), where multi-country publications indicate greater international engagement (46). Emerging contributors from the Middle East particularly Saudi Arabia, demonstrate notable international integration relative to their output, with 36.3% of publications involving cross-border collaboration (47–51). Their increasing visibility in citations and average impact may reflect broader regional efforts to strengthen HPE research capacity and expand global partnerships. Similarly, Asian countries such as China, Korea, and Japan are increasingly connected within global collaboration networks, signaling diversification of scholarly influence beyond traditional Western hubs (52).

At the institutional level, although the University of Copenhagen ranks as the most productive institution, North American institutions collectively dominate research contributions on the use of XR technology in HPE. This pattern likely reflects longstanding investment in educational research infrastructure, the availability of dedicated HPE centers, and strong funding ecosystems. Canadian institutions are also well represented, and together, the United States and Canada account for a large proportion of global output, consistent with prior bibliometric studies indicating that North America remains the most productive hub in HPE research (35, 53). Institutions in the United Kingdom also feature prominently, aligning with national priorities in competency-based HPE and stronger integration of educational scholarship into academic promotion pathways. Beyond North America and Europe, contributions from Asia-Pacific institutions such as the National University of Singapore, Shanghai Jiao University, and Taipei Medical University suggest expanding global leadership in XR-related HPE research. However, the limited representation of institutions from Africa, Latin America, and large parts of Asia highlights persistent geographic inequities in HPE scholarship (54). Strengthening institutional partnerships and research capacity in these underrepresented regions will be essential to ensure that XR innovation is informed by more globally inclusive perspectives and contextually relevant educational needs.

The rapid rise of China alongside longstanding US leadership may signal a partial shift away from historically Western-centric dominance; however, it also risks re-concentrating influence within a small number of highly resourced systems, potentially marginalizing perspectives from other East and Southeast Asian and LMIC contexts (53, 55). Similarly, the prominence of a limited group of elite institutions may shape research agendas, privileging questions, methods, and implementation models that reflect their resource-rich environments (53). These patterns are consistent with broader evidence of Global North dominance and under-representation of LMIC voices in health professions education scholarship and underscore the need for more distributed, cross-regional collaboration to avoid interpretive bias in how XR’s educational value and feasibility are understood globally (53, 55).

### Authorship patterns and scholarly influence

The analysis of authorship and citation patterns in XR Simulation-based HPE research highlights both broad participation and concentrated intellectual influence, a distribution commonly observed in HPE scholarship (56). In this dataset, XR research initially developed through a small group of highly productive authors and teams, followed by broader expansion after 2015; however, productivity remained concentrated, led by Konge L, with sustained contributions from Ahmed K and Andersen S.A.W. Such concentration is typical of emerging technology-based fields where early progress depends on specialized infrastructure and collaborative networks (46), and the subsequent growth in active authors and citations suggests a shift toward a more established area of simulation-based HPE scholarship. Despite rising publication volume, citations remain anchored to a relatively small body of pioneering work, reflecting both the increasing legitimizing role of citations (57), and cumulative advantage and citation aging processes (58, 59). Meanwhile, newer publications though methodologically relevant, are often niche and application-specific, limiting their citation reach. The coexistence of long-standing leaders and newer contributors signals a maturing and diversifying field (53, 60). Future advancement will depend on stronger cross-regional collaboration and more rigorous, transferable study designs as the literature shifts toward empirical studies and evidence syntheses.

### Influential journals and publication sources

The distribution of publication sources suggests that XR simulation-based HPE scholarship is disseminated across simulation, procedural training, digital health, and broader health professions education outlets, consistent with bibliometric patterns observed in emerging technology-driven medical research fields (61). The prominence of *Clinical Simulation in Nursing* indicates that XR-based simulation is strongly represented within nursing education, where simulation is widely embedded for competency development and patient safety (62). This finding suggests that the scope of XR simulation-based HPE research extends beyond physician-focused training. In parallel, the strong representation of surgical journals such as the *Journal of Surgical Education* and *Surgical Endoscopy and Other Interventional Techniques* implies that XR research is particularly active in procedural and minimally invasive simulation. Although learner stage cannot be directly determined from bibliometric data, the dominance of surgical and specialty outlets is consistent with a substantial focus on postgraduate and specialist training contexts, where simulation is frequently used to support technical skill acquisition and progression toward independent practice (63, 64). Conversely, the presence of general education journals such as *BMC Medical Education* suggests continued engagement in undergraduate and early postgraduate training, where XR is more likely to be applied to foundational learning needs such as anatomy and introductory clinical skills. The inclusion of digital health and informatics journals further reinforces XR’s interdisciplinary positioning and highlights the relevance of implementation, usability, and technology integration as ongoing research priorities. Based on analysis of journal impact, the distribution demonstrates that high-impact contributions to XR in simulation-based HPE arise from both specialized simulation and medical education journals as well as high-ranking surgical and technical publications (65). Collectively, these findings underscore the interdisciplinary and multi-professional scope of XR scholarship within HPE.

### Foundational literature and citation patterns

The analysis of citation patterns indicates that the intellectual foundations of XR simulation-based HPE remain strongly anchored in influential simulation scholarship while progressively transitioning toward XR-specific evidence (66). Highly cited works such as Lateef (67) and Hamstra et al. (68) continue to function as enduring reference points because they provide broadly transferable conceptual frameworks for simulation design, fidelity, and outcomes evaluation (67, 68). At the same time, the emergence of more recent highly cited work with stronger citation rates per year suggests that empirical XR applications are increasingly shaping the field, particularly within procedural and specialty-driven contexts. Importantly, because the exported dataset did not include cited-reference data (as reflected by “Cited References = 0” in Table 1), the present analysis cannot support reference-network approaches such as co-citation mapping or bibliographic coupling. Therefore, interpretations of intellectual foundations are derived from citation-based indicators rather than structural analysis of reference linkages. Future bibliometric studies incorporating complete cited-reference data would allow more robust mapping of thematic clustering and knowledge evolution within XR simulation-based HPE.

### Emerging themes and research directions

Keyword and thematic analysis indicate that XR simulation-based HPE research is thematically structured around immersive technology and simulation-related terminology. The frequent occurrence of terms such as *virtual reality*, *augmented reality*, *simulation training*, and *clinical competence* reflects their prominence within indexed literature, consistent with prior integrative and systematic reviews of XR in medical and nursing education (10, 30, 69). At the same time, keywords related to *nursing education*, *internship and residency*, and *curriculum* suggest that XR scholarships extend beyond surgical specialties and into broader health professions education contexts (69). The persistence of human-focused indexing terms reflects database conventions rather than conceptual shifts and was interpreted with caution.

The emergence of COVID-19 as a recurring keyword highlights how the pandemic was associated with increased attention to XR adoption during disruption to in-person training and reduced clinical access (10, 70). Subsequent literature suggests that XR integration continued beyond the acute pandemic phase, indicating sustained incorporation into evolving models of HPE (70).

Thematic evolution analysis demonstrates continuity rather than disruption, across two time periods. Core descriptors, particularly “simulation” and human-related terms, remained prominent in both 2010–2021 and 2022–2025. This persistence indicates stability in the conceptual vocabulary of the field rather than substantial thematic displacement over time. While “virtual reality” appeared as a distinct thematic cluster in the earlier period, its later positioning within a broader “human-virtual reality-augmented reality” basic cluster suggests structural convergence of immersive modality terminology within the mapped literature, as reflected in shifts in centrality and density positioning (71). Strategic thematic mapping further demonstrates that procedural and surgery related themes occupied highly central basic positions in the earlier period, whereas immersive terminology formed a central basic cluster in the later period, reinforcing the dominant role of technical and performance-based training contexts (70).

Collectively, these patterns suggest that XR-related HPE scholarship is organized around immersive and simulation-based terminology alongside broader pedagogical adaptation across diverse health professions disciplines. The comparatively lower centrality of keywords related to non-technical skills, interprofessional education, and curriculum integration suggests that these areas remain less represented within the indexed literature.

### Limitations

This bibliometric study has several limitations. Although the search was conducted across PubMed, Scopus, and Web of Science, some relevant publications may have been missed due to indexing inconsistencies and variation in terminology. Records for 2025 represent a partial year because the search was conducted on July 29, 2025; therefore, publication and citation trends for 2025 may appear artificially lower than complete years. The restriction to English-language publications may bias findings toward English-language journals and countries with high English publication output, potentially underrepresenting XR simulation-related HPE scholarship published in other languages. In addition, institutional and country productivity estimates may be affected by inconsistencies in affiliation reporting, which can fragment institutional outputs. Citation-based indicators are also subject to citation lag, meaning that recently published high-quality work may be underrepresented in impact analyses. Citation counts were obtained from more than one database (primarily WoS and Scopus), which differ in citation indexing. PubMed-only records also lack citation data in the exported format. As a result, citation-based results in this review are best interpreted as indicating relative patterns and trends rather than precise cross-database comparisons. Finally, the exported dataset did not include cited-reference dataset, precluding reference-network analyses such as co-citation mapping and bibliographic coupling; therefore, interpretations of intellectual structure are based on citation metrics rather than structural reference linkages.

### Future directions

Future research should address citation lag by conducting longitudinal updates of bibliometric analyses to capture the evolving impact of recent high-quality publications. Additionally, incorporating cited reference data would enable a deeper exploration of the intellectual structure of XR research in HPE, including mapping knowledge flows and interconnections across disciplines. Expanding cross-database comparisons and integrating alternative metrics (e.g., altmetrics) may further enrich understanding of both scholarly and practical influence in this rapidly developing field. Sustained international collaboration and stronger inclusion of underrepresented regions will be essential for building a more globally representative evidence base to inform policy and practice in HPE.

## Conclusion

This bibliometric analysis provides cross-database mapping of extended reality (XR) research in simulation-based HPE from 2010 to 2025. By integrating publications from Web of Science, Scopus, and PubMed, the study indicates that XR simulation-based HPE has transitioned from a period of emergent innovation to a sustained academic field. Steady growth in publication output and global participation led by the United States and university-based research centers reflects the growing integration of XR scholarship into mainstream simulation-based HPE research. At the authorship level, despite diversification of author networks, global collaboration remains limited in several high-producing countries, highlighting the need to strengthen cross-regional partnerships and improve generalizability. Thematically, “virtual reality,” “simulation,” and “augmented reality” continue to shape the research landscape. Future work should prioritize expanding XR scholarship in underrepresented regions, broadening applications beyond procedural training to include non-technical and interprofessional skills, and advancing rigorous, multicenter, theory-informed designs across learner stages. Strategic rebalancing toward broader competencies, diverse learner populations, and global partnerships will be important to strengthen the relevance and applicability of XR-related HPE research.

## Data Availability

The original contributions presented in the study are included in the article/Supplementary material, further inquiries can be directed to the corresponding author.
