# Supplementary material for: The use of the extended reality technologies in simulation-based health professions education: a bibliometric analysis
Source: Front Med (Lausanne). 2026 Apr 13;13:1787542. doi: 10.3389/fmed.2026.1787542 (PMC13111093; doi:10.3389/fmed.2026.1787542)
Supplement: Supplementary file 1 [file Data_Sheet_1.pdf]

Supplementary Material

Supplementary Material 1. A detailed description of the search terms and strategy			
**Source and coverage**	**Search string**	**Results**	**notes**
**PubMed (NLM)** **Coverage:** From database inception-2025-07-29	**((((((((((((((((((((((((("Education, Medical"[MeSH Terms]) OR ("Medical Education"[Title/Abstract])) OR ("Education, Medical, Continuing"[MeSH Terms])) OR ("Education, Medical, Graduate"[MeSH Terms])) OR ("Education, Medical, Undergraduate"[MeSH Terms])) OR ("Education, Premedical"[MeSH Terms])) OR ("Education, Public Health Professional"[MeSH Terms])) OR ("Fellowships and Scholarships"[MeSH Terms])) OR ("Internship and Residency"[MeSH Terms])) OR ("Health Professions Education"[Title/Abstract])) OR ("Medical Instruction"[Title/Abstract])) OR ("Medical Program"[Title/Abstract])) OR ("Medical Teaching"[Title/Abstract])) OR ("Medical Training"[Title/Abstract])) OR ("Training, Medical"[Title/Abstract])) OR ("Undergraduate Medical Education"[Title/Abstract])) OR ("Clinical Education"[Title/Abstract])) OR ("Curriculum Development"[MeSH Terms])) OR ("Medicine"[Title/Abstract])) OR ("Nursing"[Title/Abstract])) OR ("Physiotherapy"[Title/Abstract])) OR ("Chiropractic"[Title/Abstract])) OR ("Surgery"[Title/Abstract])) OR ("Radiology"[Title/Abstract])) OR ("Anatomy"[Title/Abstract])) AND ((((((((((((((("Simulation Training"[MeSH Terms]) OR ("Education, Medical"[MeSH Terms])) OR ("Computer Simulation"[MeSH Terms])) OR ("Manikins"[MeSH Terms])) OR ("Teaching"[MeSH Terms])) OR ("Medical Simulation"[Title/Abstract])) OR ("Clinical Simulation"[Title/Abstract])) OR ("Healthcare Simulation"[Title/Abstract])) OR ("Simulation-based Medical Education"[Title/Abstract])) OR ("Simulation Training"[Title/Abstract])) OR ("Simulated Learning Environment"[Title/Abstract])) OR ("Patient Simulations"[Title/Abstract])) OR ("Simulation,**	1,542	All search terms are searched in the field: [Title/Abstract] and in MeSH (when available). filters or English language, articles and reviews **Years 2010-2025**

	**Patient"[Title/Abstract])) OR ("Simulations, Patient"[Title/Abstract])) OR ("Clinical skills training"[Title/Abstract])) OR ("simulators"[Title/Abstract]))) AND (((((((((((((((((("Augmented Reality"[MeSH Terms]) OR ("Augmented Reality"[Title/Abstract])) OR ("Extended Reality"[Title/Abstract])) OR ("Virtual Reality"[MeSH Terms])) OR ("Virtual Reality"[Title/Abstract])) OR ("Mixed Reality"[Title/Abstract])) OR ("Augmented Virtuality"[Title/Abstract])) OR ("Immersive Technology"[Title/Abstract])) OR ("Immersive Environments"[Title/Abstract])) OR ("Immersive Learning"[Title/Abstract])) OR ("XR in Healthcare"[Title/Abstract])) OR ("Head-Mounted Display"[Title/Abstract])) OR ("3D Simulation Environments"[Title/Abstract])) OR ("Serious Games"[Title/Abstract])) OR ("Holographic Simulation"[Title/Abstract])) OR ("Spatial Computing"[Title/Abstract])) OR ("Serious Games"[MeSH Terms])) OR ("XR"[Title/Abstract]))** Filters: **Bibliography, Biography, Case Reports, Clinical Conference, Clinical Study, Clinical Trial, Clinical Trial Protocol, Clinical Trial, Phase I, Clinical Trial, Phase II, Clinical Trial, Phase III, Clinical Trial, Phase IV, Clinical Trial, Veterinary, Consensus Development Conference, Consensus Development Conference, NIH, Meta-Analysis, Observational Study, Randomized Controlled Trial, Review, Scientific Integrity Review, Scoping Review, Systematic Review, Technical Report, English, Humans, from 2010 - 2025**		
**Scopus (Elsevier)** **Coverage:** From database inception-2025-07-29	**(TITLE-ABS-KEY ("Medical Education") OR TITLE-ABS-KEY ("Education, Medical") OR TITLE-ABS-KEY ("Education, Medical, Continuing") OR TITLE-ABS-KEY ("Education, Medical, Graduate") OR TITLE-ABS-KEY ("Education, Medical, Undergraduate") OR TITLE-ABS-KEY ("Education, Premedical") OR TITLE-ABS-KEY ("Education, Public Health Professional") OR TITLE-ABS-KEY ("Fellowships and Scholarships") OR TITLE-ABS-KEY ("Health Professions Education") OR TITLE-ABS-KEY ("Internship and Residency") OR TITLE-ABS-KEY ("Medical Instruction") OR TITLE-ABS-KEY ("Medical Program") OR TITLE-ABS-KEY ("Medical Teaching") OR TITLE-ABS-KEY ("Medical Training") OR TITLE-ABS-KEY ("Training, Medical") OR TITLE-ABS-KEY ("Undergraduate Medical Education") OR TITLE-ABS-KEY ("Curriculum Development") OR TITLE-ABS-KEY ("Clinical**	592 results	All search terms are searched in the fields: “title”, “abstract” and “keywords” (here marked with “TITLE-ABS-

	**Education") OR TITLE-ABS-KEY ("Anatomy") OR TITLE-ABS-KEY ("Medicine") OR TITLE-ABS-KEY ("Radiology") OR TITLE-ABS-KEY ("Nursing") OR TITLE-ABS-KEY ("Surgery") OR TITLE-ABS-KEY ("Chiropractic") OR TITLE-ABS-KEY ("Physiotherapy")) AND (TITLE-ABS-KEY ("virtual reality") OR TITLE-ABS-KEY ("augmented reality") OR TITLE-ABS-KEY ("mixed reality") OR TITLE-ABS-KEY ("extended reality") OR TITLE-ABS-KEY ("immersive technology") OR TITLE-ABS-KEY ("immersive environments") OR TITLE-ABS-KEY ("immersive Learning") OR TITLE-ABS-KEY ("XR in healthcare") OR TITLE-ABS-KEY ("head mounted display") OR TITLE-ABS-KEY ("3D simulation environments") OR TITLE-ABS-KEY ("serious games") OR TITLE-ABS-KEY ("holographic simulation") OR TITLE-ABS-KEY ("spatial computing") OR TITLE-ABS-KEY ("XR")) AND (TITLE-ABS-KEY ("Medical Simulation") OR TITLE-ABS-KEY ("Clinical Simulation") OR TITLE-ABS-KEY ("Healthcare Simulation") OR TITLE-ABS-KEY ("Simulation-based Medical Education") OR TITLE-ABS-KEY ("Simulation Training") OR TITLE-ABS-KEY ("Simulated Learning Environment") OR TITLE-ABS-KEY ("Patient Simulations") OR TITLE-ABS-KEY ("Simulation, Patient") OR TITLE-ABS-KEY ("Simulations, Patient") OR TITLE-ABS-KEY ("Clinical Skills Training") OR TITLE-ABS-KEY ("Simulators")) AND PUBYEAR > 2009 AND PUBYEAR < 2026 AND (LIMIT-TO (SUBJAREA , "MEDI") OR LIMIT-TO (SUBJAREA , "HEAL") OR LIMIT-TO (SUBJAREA , "SOCI") OR LIMIT-TO (SUBJAREA , "NURS") OR LIMIT-TO (SUBJAREA , "NEUR") OR LIMIT-TO (SUBJAREA , "ENVI")) AND (LIMIT-TO (DOCTYPE , "ar") OR LIMIT-TO (DOCTYPE , "re") OR LIMIT-TO (DOCTYPE , "cp") OR LIMIT-TO (DOCTYPE , "cr")) AND (LIMIT-TO (LANGUAGE , "English")) AND (EXCLUDE (EXACTKEYWORD , "Humans") OR EXCLUDE (EXACTKEYWORD , "Priority Journal") OR EXCLUDE (EXACTKEYWORD , "Young Adult") OR EXCLUDE (EXACTKEYWORD , "Adult") OR EXCLUDE (EXACTKEYWORD , "Middle Aged") OR EXCLUDE (EXACTKEYWORD , "Psychology"))**		KEY”) filters or limitations English language, articles and reviews<br><br>**Years 2010-2025**

	OR EXCLUDE (EXACTKEYWORD , "Animals") OR EXCLUDE (EXACTKEYWORD , "Nonhuman") OR EXCLUDE (EXACTKEYWORD , "Animal") OR EXCLUDE (EXACTKEYWORD , "Aged") OR EXCLUDE (EXACTKEYWORD , " Normal Human"))		
**Web of Science-Core Collection (Clarivate)**<br>**Coverage:**<br>From database inception-2025-07-29	**Query 1**<br>**(((((((((((((((((((((((TS=("Medical Education")) OR TS=("Education, Medical")) OR TS=("Education, Medical, Continuing")) OR TS=("Education, Medical, Graduate")) OR TS=("Education, Medical, Undergraduate")) OR TS=("Education, Premedical")) OR TS=("Education, Public Health Professional")) OR TS=("Fellowships and Scholarships")) OR TS=("Health Professions Education")) OR TS=("Internship and Residency")) OR TS=("Medical Instruction")) OR TS=("Medical Program*")) OR TS=("Medical Teaching")) OR TS=("Medical Training")) OR TS=("Training, Medical")) OR TS=("Undergraduate Medical Education")) OR TS=("Curriculum Development")) OR TS=("Clinical Education")) OR TS=("anatomy")) OR TS=("Surgery")) OR TS=("Medicine")) OR TS=("Physiotherapy")) OR TS=("Radiology")) OR TS=("Chiropractic")) OR TS=("Nursing")**<br>**Query 2**<br>**(((((((((TS=("Medical Simulation")) OR TS=("Clinical Simulation")) OR TS=("Healthcare Simulation")) OR TS=("Simulation-based Medical Education")) OR TS=("Simulation Training")) OR TS=("Simulated Learning Environment")) OR TS=("Patient Simulations")) OR TS=("Simulation, Patient")) OR TS=("Simulations, Patient")) OR TS=("Clinical skills training")) OR TS=("simulators")**<br>**Query 3**<br>**(((((((((((((TS=("Extended Reality")) OR TS=("Augmented Reality")) OR TS=("Virtual Reality")) OR TS=("Mixed Reality")) OR TS=("Augmented Virtuality")) OR TS=("Immersive Technology")) OR TS=("Immersive Environments")) OR TS=("Immersive Learning")) OR TS=("XR in Healthcare")) OR TS=("Head-Mounted Display*")) OR TS=("3D Simulation Environment*")) OR TS=("Serious Game*")) OR TS=("Holographic Simulation")) OR TS=("Spatial Computing")) OR TS=("XR")**<br>**#1 AND #2 AND #1 AND #2 AND #3** and **2010** or **2011** or **2012** or **2013** or **2014** or **2025** or **2024** or **2023** or **2022** or **2021** or **2020** or **2019** or **2018** or **2017** or **2016** or **2015** (Publication Years) and **Article** or **Review Article** or **Proceeding**	expand_moreShow editions<br>970<br>**970**	All search terms are searched in the field: "Topic" (including title, abstract and author supplied keywords, here marked with "TOPIC"). filters or limitations English language, articles and reviews<br>**Years 2010-2025**

	**Paper** (Document Types) and **English** (Languages) and **Surgery** or **Nursing** or **Health Care Sciences Services** or **General Internal Medicine** or **Neurosciences Neurology** or **Research Experimental Medicine** or **Anatomy Morphology** or **Radiology Nuclear Medicine Medical Imaging** or **Robotics** (Research Areas)<br><br>https://www.webofscience.com/wos/woscc/summary/6ed5464e-b0ca-4d46-935c-25e823fd6276-016e9beb67/relevance/1		
**Total no. references identified**	**3,121**		
**Total no. unique references identified after automatic de-duplication in Bibliometrix**	**2,789**		
**duplicate identified**	**332**		

**Supplementary Material 2.** Most Relevant Affiliations		
No.	Affiliation	Articles
**1**	UNIVERSITY OF COPENHAGEN	287
**2**	UNIVERSITY OF LONDON	236
**3**	HARVARD UNIVERSITY	213
**4**	UNIVERSITY OF TORONTO	181
**5**	IMPERIAL COLLEGE LONDON	175
**6**	UNIVERSITY SYSTEM OF OHIO	159
**7**	UNIVERSITY OF CINCINNATI	131
**8**	MCGILL UNIVERSITY	125
**9**	MAYO CLINIC	120
**10**	WESTERN UNIVERSITY	109
**11**	JOHNS HOPKINS UNIVERSITY	108
**12**	NATIONAL UNIVERSITY OF SINGAPORE	95
**13**	KAROLINSKA INSTITUTET	91
**14**	UNIVERSITY OF HEIDELBERG	83
**15**	SHANGHAI JIAO TONG UNIVERSITY	83
**16**	UNIVERSITY OF TURIN	76
**17**	TAIPEI MEDICAL UNIVERSITY	69

**Supplementary Material 3.** Annual Scientific Production		
**No.**	**Year**	**Articles**
1	**2010**	54
2	**2011**	83
3	**2012**	74
4	**2013**	92
5	**2014**	118
6	**2015**	82
7	**2016**	106
8	**2017**	111
9	**2018**	136
10	**2019**	185
11	**2020**	229
12	**2021**	241
13	**2022**	257
14	**2023**	300
15	**2024**	406
16	**2025**	315

Supplementary Material 4. Average Citation per Year

Year	Mean Total Citation per Article	Number of Citation Per Year	Mean Total Citation per Year	CitableYears
**2010**	47.56	54	2.97	16
**2011**	19.80	83	1.32	15
**2012**	21.50	74	1.54	14
**2013**	33.30	92	2.56	13
**2014**	24.47	118	2.04	12
**2015**	23.07	82	2.10	11
**2016**	21.97	106	2.20	10
**2017**	16.78	111	1.86	9
**2018**	16.46	136	2.06	8
**2019**	12.79	185	1.83	7
**2020**	10.41	229	1.74	6
**2021**	8.59	241	1.72	5
**2022**	4.98	257	1.25	4
**2023**	3.69	300	1.23	3
**2024**	1.38	406	0.69	2
**2025**	0.12	315	0.12	1

**Supplementary Material 5.** Sources' Local Impact by H Index		
**No.**	**Source**	**H index**
1	SURGICAL ENDOSCOPY AND OTHER INTERVENTIONAL TECHNIQUES	27
2	JOURNAL OF SURGICAL EDUCATION	24
3	CLINICAL SIMULATION IN NURSING	22
4	SIMULATION IN HEALTHCARE-JOURNAL OF THE SOCIETY FOR SIMULATION IN HEALTHCARE	14
5	JMIR SERIOUS GAMES	13
6	WORLD NEUROSURGERY	12
7	NEUROSURGERY	11
8	INTERNATIONAL JOURNAL OF MEDICAL ROBOTICS AND COMPUTER ASSISTED SURGERY	10
9	NURSE EDUCATION TODAY	10
10	AMERICAN JOURNAL OF SURGERY	9
11	ANNALS OF SURGERY	9
12	ARTHROSCOPY-THE JOURNAL OF ARTHROSCOPIC AND RELATED SURGERY	9
13	INTERNATIONAL JOURNAL OF COMPUTER ASSISTED RADIOLOGY AND SURGERY	9
14	JOURNAL OF SURGICAL RESEARCH	9
15	SURGERY	9

Supplementary Material 6. Most Global Cited Documents

Paper	Title	DOI	Total Citations	Total Citation per Year	Normalized Total Citation
LATEEF F, 2010, J EMERG TRAUMA SHOCK	**1.1.1 Simulation-based learning: Just like the real thing**	10.4103/0974-2700.70743	563	35.1875	11.83878505
VAN H P D, 2010, BRIT J SURG	**1.1.2 Objective assessment of technical surgical skills**	10.1002/bjs.7115	376	23.5	7.906542056
HAMSTRA S J, 2014, ACAD MED	**1.1.3 Reconsidering fidelity in simulation-based training**	10.1097/ACM.0000000000000130	363	30.25	14.83685487
LI L, 2017, AM J TRANSL RES	**1.1.4 Application of virtual reality technology in clinical medicine**	PMC5622235	320	35.55555556	19.06602254
KOTSIS S V, 2013, PLAST RECONSTR SURG	**2 Application of the "see one, do one, teach one" concept in surgical training**	10.1097/PRS.0b013e318287a0b3	271	20.84615385	8.137075718

ZENDEJAS B, 2013, ANN SURG	**3 State of the evidence on simulation-based training for laparoscopic surgery: a systematic review**	10.1097/SLA.0b013e318288c40b	266	20.46153846	7.98694517
COOK D A, 2013, ACAD MED	**4 Mastery learning for health professionals using technology-enhanced simulation: a systematic review and meta-analysis**	10.1097/ACM.0b013e31829a365d	257	19.76923077	7.716710183
ZENDEJAS B, 2013, J GEN INTERN MED	**4.1.1 Patient outcomes in simulation-based medical education**	10.1007/s11606-012-2264-5	233	17.92307692	6.996083551
ZHU E, 2014, PEERJ	Augmented reality in healthcare education: an integrative review.	10.7717/peerj.469	222	18.5	9.073779009
BADASH I, 2016, ANN TRANSL MED	Innovations in surgery simulation: a review of past, current and future techniques.	10.21037/atm.2016.12.24	217	21.7	9.876341778

**Supplementary Material 7.** Authors' Local Impact by H Index							
**No.**	**Author**	**H_index**	**g_index**	**m_index**	**TC**	**NP**	**PY_start**
1	KONGE L	18	31	1.384615385	987	53	2013
2	AHMED K	16	28	1	811	32	2010
3	DASGUPTA P	15	23	1.153846154	560	26	2013
4	AYDIN A	12	20	1	545	20	2014
5	ANDERSEN S A W	11	16	1.1	522	16	2016
6	BJERRUM F	10	19	0.714285714	517	19	2012
7	DE S	10	16	0.625	268	25	2010
8	JONES D B	10	13	0.714285714	440	13	2012
9	SORENSEN M S	10	13	0.909090909	382	13	2015
19	AGGARWAL R	9	21	0.5625	518	21	2010

**Supplementary Material 8.** Country scientific Production		
**No.**	**Country**	**Frequency**
1	USA	2171
2	CHINA	1221

3	CANADA	766
4	ITALY	619
5	GERMANY	580
6	FRANCE	515
7	UK	501
8	SPAIN	454
9	AUSTRALIA	366
10	DENMARK	352
11	NETHERLANDS	340
12	JAPAN	234
13	SOUTH KOREA	192
14	BRAZIL	183
15	TURKEY	176
16	SWITZERLAND	155
17	SWEDEN	147
18	IRELAND	138
19	INDIA	130
20	SINGAPORE	126
21	BELGIUM	124
22	NORWAY	112

23	GREECE	96
24	IRAN	95
25	AUSTRIA	92
26	SAUDI ARABIA	83
27	POLAND	77
28	PAKISTAN	68
29	PORTUGAL	63
30	ISRAEL	49
31	CZECH REPUBLIC	41
32	ROMANIA	40
33	FINLAND	36
34	EGYPT	32
35	COLOMBIA	30
36	MEXICO	26
37	MALAYSIA	24
38	HUNGARY	22
39	NEW ZEALAND	22
40	QATAR	21
41	CHILE	20
42	INDONESIA	18

43	SERBIA	17
44	PERU	16
45	RUSSIA	13
46	NIGERIA	12
47	CYPRUS	11
48	SLOVENIA	11
49	MOROCCO	8
50	THAILAND	8
51	OMAN	7
52	ARGENTINA	6
53	PHILIPPINES	6
54	SOUTH AFRICA	6
55	UNITED ARAB EMIRATES	6
56	VIETNAM	6
57	CROATIA	5
58	JORDAN	5
59	KENYA	5
60	LUXEMBOURG	5
61	SLOVAKIA	5
62	ECUADOR	4

63	LEBANON	4
64	MALTA	4
65	NEPAL	4
66	TUNISIA	3
67	BANGLADESH	2
68	BARBADOS	2
69	BULGARIA	2
70	KUWAIT	2
71	LIBERIA	2
72	NORTH MACEDONIA	2
73	RWANDA	2
74	UKRAINE	2
75	VENEZUELA	2
76	ARMENIA	1
77	BENIN	1
78	BOLIVIA	1
79	CAMBODIA	1
80	COSTA RICA	1
81	GEORGIA	1
82	ICELAND	1

83	IRAQ	1
84	LATVIA	1
85	LITHUANIA	1
86	MALAWI	1
87	MONACO	1
88	YEMEN	1

Supplementary Material 9. GCC Country scientific Production

No.	Country	Frequency
1	SAUDI ARABIA	83
2	QATAR	21
3	OMAN	7
4	UNITED ARAB EMIRATES	6
5	KUWAIT	2

Supplementary Material 10. Country Production Over Time

	Canada	China	Germany	USA	Italy
**2010**	4	3	1	52	0

**2011**	12	6	5	101	7
**2012**	30	7	6	135	7
**2013**	76	12	13	215	9
**2014**	121	13	20	293	12
**2015**	135	13	28	350	22
**2016**	161	26	62	420	30
**2017**	210	48	85	468	35
**2018**	238	66	114	579	62
**2019**	279	125	154	646	91
**2020**	368	209	178	854	123
**2021**	431	309	222	1079	180
**2022**	512	457	321	1295	273
**2023**	579	692	394	1551	362
**2024**	692	971	616	1932	531
**2025**	766	1221	580	2171	619

Supplementary Material 11. Most Cited Countries

No.	Country	Total Citation	Average Article Citations
1	USA	8909	25.10

2	CANADA	3177	31.50
3	UNITED KINGDOM	3120	25.20
4	CHINA	1502	17.90
5	DENMARK	1375	29.90
6	NETHERLANDS	1243	18.80
7	AUSTRALIA	1227	31.50
8	SINGAPORE	1160	82.90
9	GERMANY	906	17.10
10	FRANCE	812	17.70

Supplementary Material 12. Affiliations' Production over Time

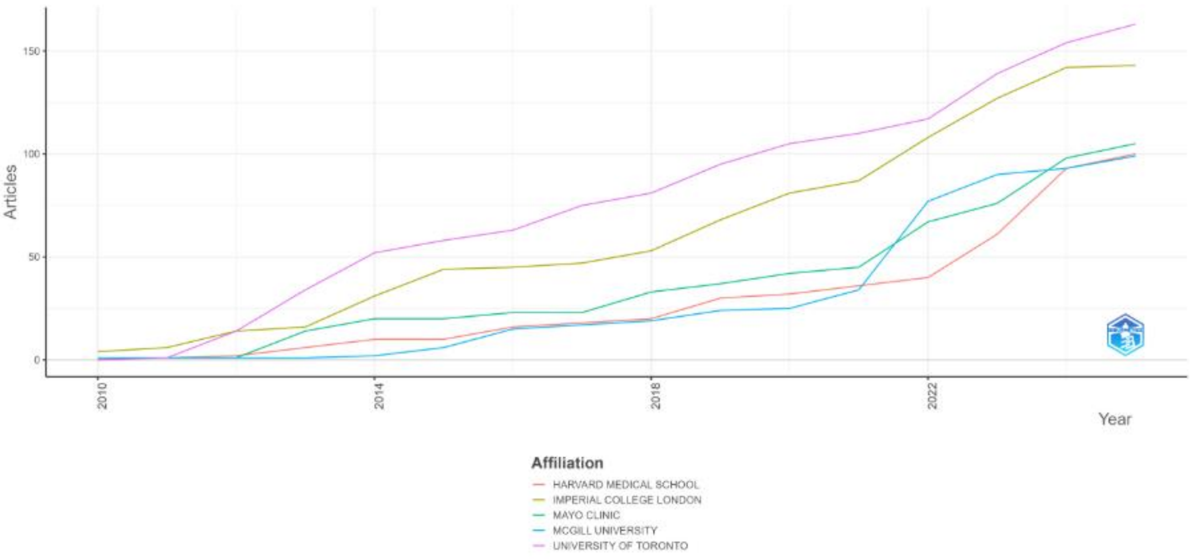

Supplementary Material 13. Most Relevant Sources

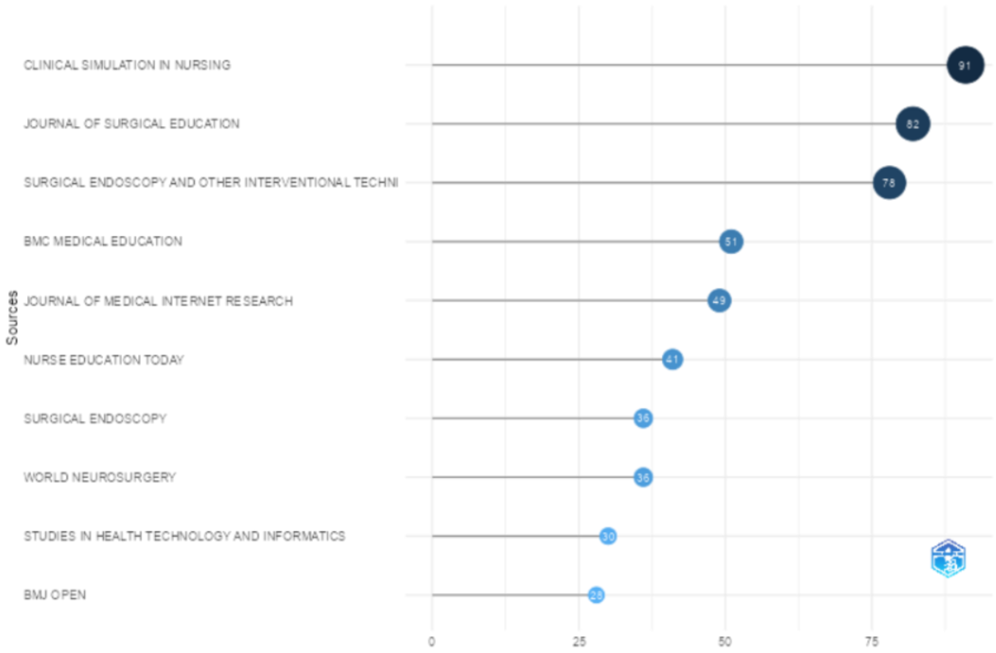

Supplementary Material 14. Sources; Local Impact by H Index

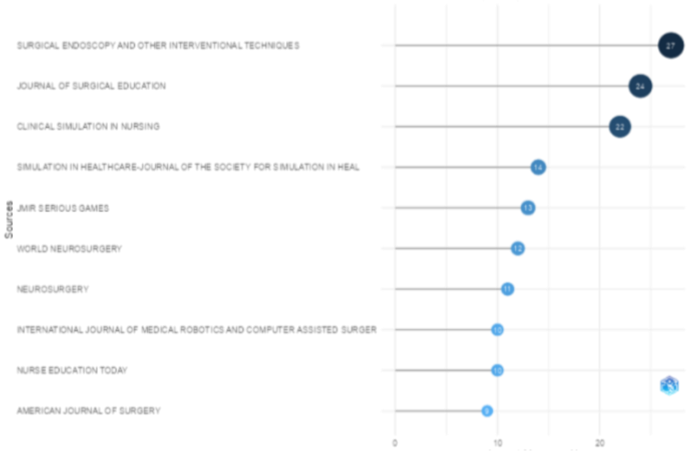

**Supplementary Material 15.** Sources Production over Time

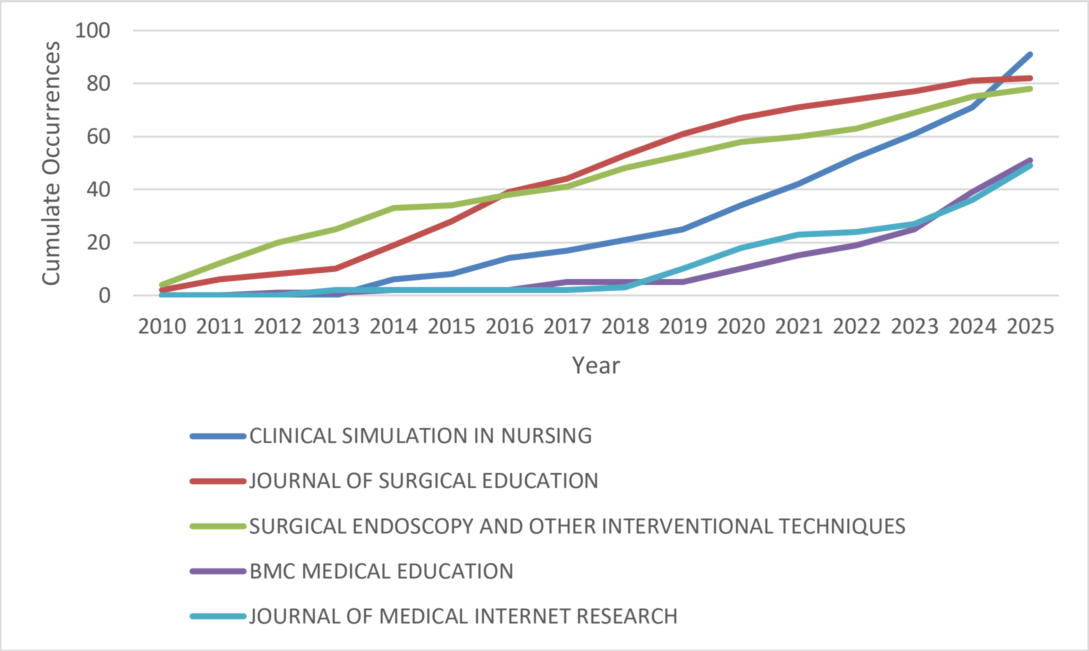

**Supplementary Material 16.** Authors' Local Impact by H Index

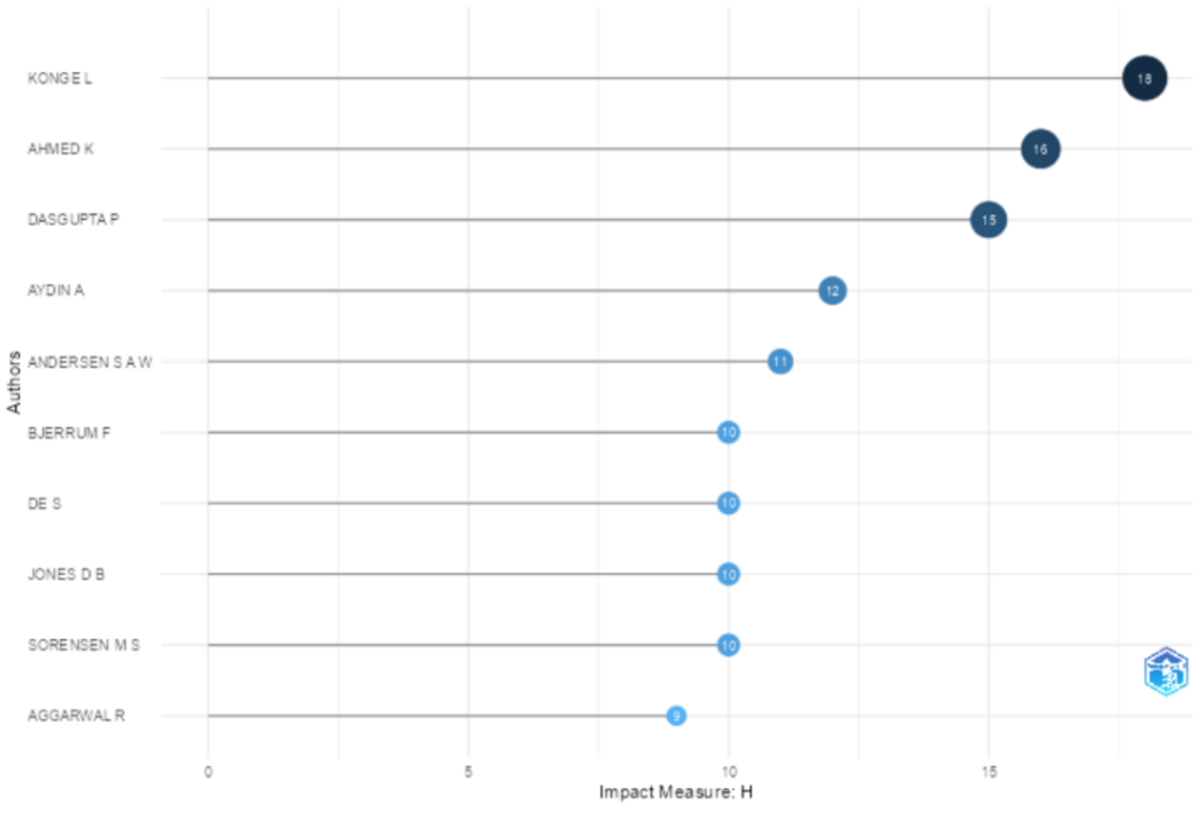

Supplementary Material 17. Authors' Collaboration Network

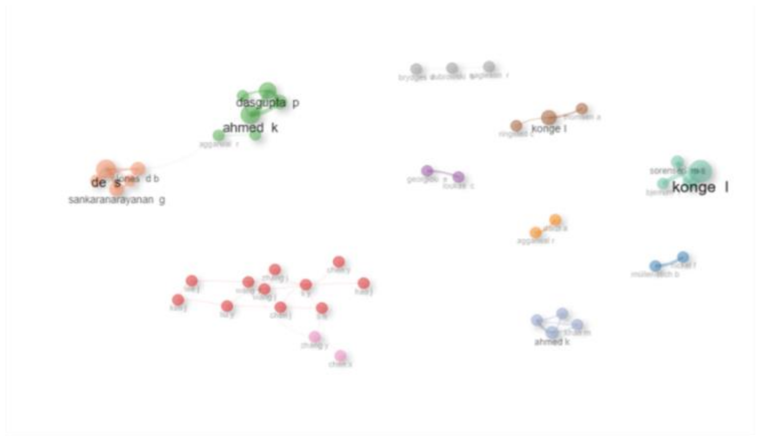

Supplementary Material 18. GCC Country Scientific Production

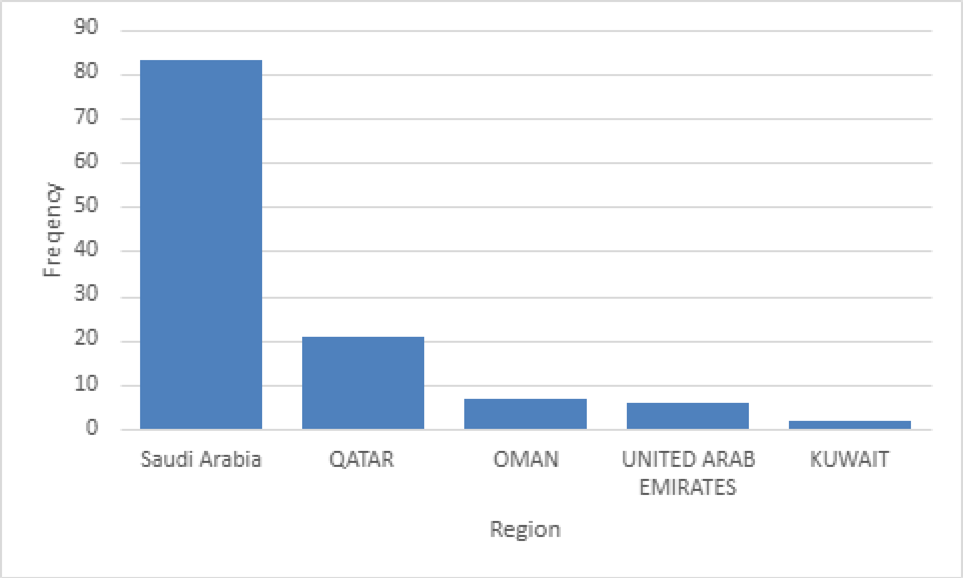

Supplementary Material 19. Country Production over Time

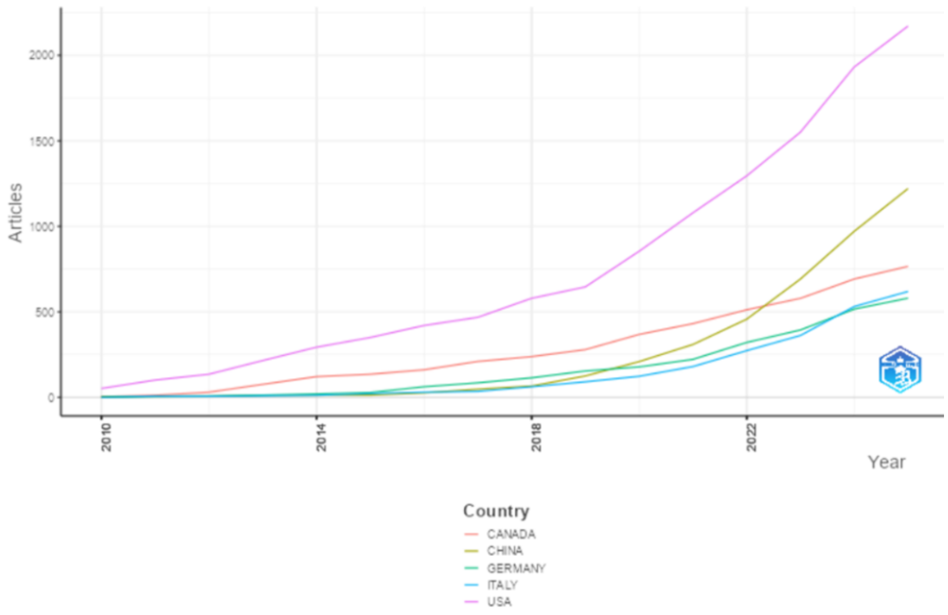

Supplementary Material 20. Most Cited Countries

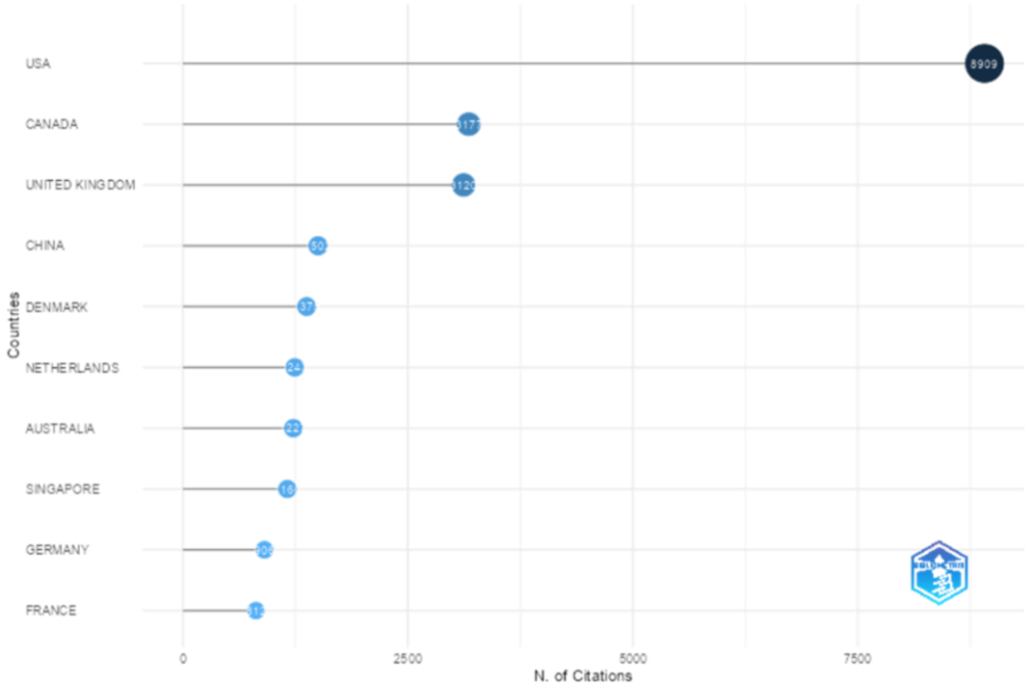

Supplementary Material 21. Most Global Cited Documents

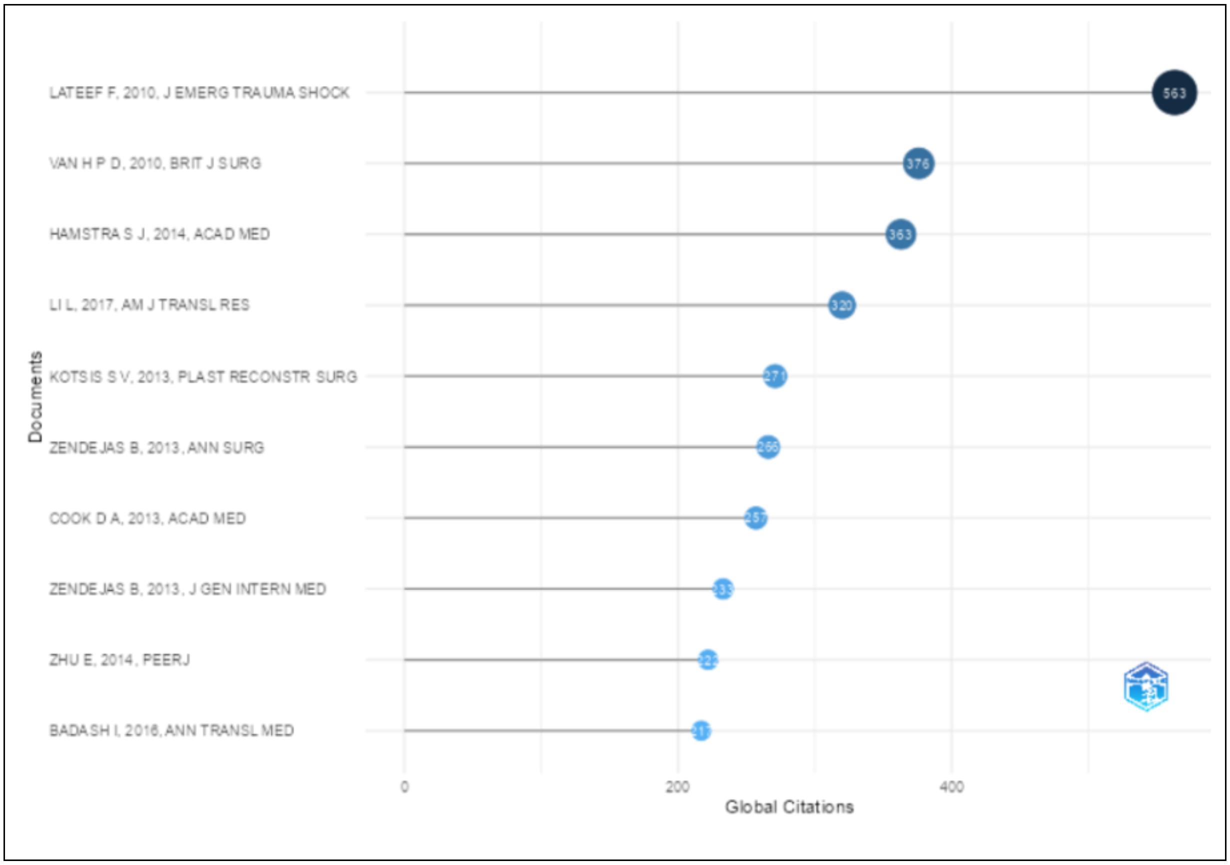
LATEEF F, 2010, J EMERG TRAUMA SHOCK
VAN H P D, 2010, BRIT J SURG
HAMSTRA S J, 2014, ACAD MED
LI L, 2017, AM J TRANSL RES
KOTSIS S V, 2013, PLAST RECONSTR SURG
ZENDEJAS B, 2013, ANN SURG
COOK D A, 2013, ACAD MED
ZENDEJAS B, 2013, J GEN INTERN MED
ZHU E, 2014, PEERJ
BADASH I, 2016, ANN TRANSL MED
Documents
563
376
363
320
271
266
257
233
0
200
400
Global Citations
